# A novel small molecule that kills a subset of MLL-rearranged leukemia cells by inducing mitochondrial dysfunction

**DOI:** 10.1038/s41388-018-0666-5

**Published:** 2019-01-22

**Authors:** Klaartje Somers, Victoria W. Wen, Shiloh M. C. Middlemiss, Brenna Osborne, Helen Forgham, MoonSun Jung, Mawar Karsa, Molly Clifton, Angelika Bongers, Jixuan Gao, Chelsea Mayoh, Newsha Raoufi-Rad, Eric P. Kusnadi, Kate M. Hannan, David A. Scott, Alan Kwek, Bing Liu, Claudia Flemming, Daria A. Chudakova, Ruby Pandher, Tim W. Failes, James Lim, Andrea Angeli, Andrei L. Osterman, Toshihiko Imamura, Ursula R. Kees, Claudiu T. Supuran, Richard B. Pearson, Ross D. Hannan, Thomas P. Davis, Joshua McCarroll, Maria Kavallaris, Nigel Turner, Andrei V. Gudkov, Michelle Haber, Murray D. Norris, Michelle J. Henderson

**Affiliations:** 1 Children’s Cancer Institute, Lowy Cancer Research Centre, UNSW, Randwick, NSW Australia; 2 Mitochondrial Bioenergetics Laboratory, School of Medical Sciences, UNSW, Randwick, NSW Australia; 3 ARC Centre of Excellence in Convergent Bio-Nano Science and Technology, Australian Centre for NanoMedicine, UNSW Australia, Sydney, NSW Australia; 4 Peter MacCallum Cancer Centre, Melbourne, VIC Australia; 5 The John Curtin School of Medical Research, The Australian National University, Canberra City, ACT Australia; 6 Sanford Burnham Prebys Medical Discovery Institute, La Jolla, CA 92037 USA; 7 ACRF Drug Discovery Centre, Children’s Cancer Institute, Lowy Cancer Research Centre, UNSW, Sydney, New South Wales Australia; 8 Neurofarba Department, University of Florence, Florence, Italy; 9 Department of Pediatrics, Kyoto Prefectural University of Medicine, Kyoto, Japan; 10 Telethon Kids Institute, University of Western Australia, Perth, WA Australia; 11 ARC Centre of Excellence in Convergent Bio-Nano Science and Technology Monash Institute of Pharmaceutical Sciences, Monash University, Clayton, VIC Australia; 12 Department of Chemistry, University of Warrick, Coventry, UK; 13 Department of Cell Stress Biology, Roswell Park Cancer Institute, Buffalo, NY USA; 14 Oncotartis, Inc., Buffalo, NY USA; 15 UNSW Centre for Childhood Cancer Research, Sydney, NSW Australia

**Keywords:** Leukaemia, Paediatric cancer

## Abstract

Survival rates for pediatric patients suffering from *mixed lineage leukemia* (*MLL*)-rearranged leukemia remain below 50% and more targeted, less toxic therapies are urgently needed. A screening method optimized to discover cytotoxic compounds selective for MLL-rearranged leukemia identified CCI-006 as a novel inhibitor of MLL-rearranged and CALM-AF10 translocated leukemias that share common leukemogenic pathways. CCI-006 inhibited mitochondrial respiration and induced mitochondrial membrane depolarization and apoptosis in a subset (7/11, 64%) of MLL-rearranged leukemia cell lines within a few hours of treatment. The unresponsive MLL-rearranged leukemia cells did not undergo mitochondrial membrane depolarization or apoptosis despite a similar attenuation of mitochondrial respiration by the compound. In comparison to the sensitive cells, the unresponsive MLL-rearranged leukemia cells were characterized by a more glycolytic metabolic phenotype, exemplified by a more pronounced sensitivity to glycolysis inhibitors and elevated HIF1α expression. Silencing of HIF1α expression sensitized an intrinsically unresponsive MLL-rearranged leukemia cell to CCI-006, indicating that this pathway plays a role in determining sensitivity to the compound. In addition, unresponsive MLL-rearranged leukemia cells expressed increased levels of *MEIS1*, an important leukemogenic MLL target gene that plays a role in regulating metabolic phenotype through HIF1α. MEIS1 expression was also variable in a pediatric MLL-rearranged ALL patient dataset, highlighting the existence of a previously undescribed metabolic variability in MLL-rearranged leukemia that may contribute to the heterogeneity of the disease. This study thus identified a novel small molecule that rapidly kills MLL-rearranged leukemia cells by targeting a metabolic vulnerability in a subset of low HIF1α/low MEIS1-expressing MLL-rearranged leukemia cells.

## Introduction

Rearrangement of the *mixed lineage leukemia* (*MLL*, *KMT2A*) gene occurs in 5–10% of acute leukemias and is especially prevalent in infant acute leukemias (up to 70% of cases) [1]. The MLL-rearranged (MLL-r) leukemia subtype is characterized by its aggressive nature, resistance to therapy and typical occurrence of early relapse, even after initially achieving complete remission, resulting in 5-year event-free survival rates of less than 50% [1, 2]. Due to the high-risk classification of MLL-r leukemia, clinical chemotherapeutic treatment protocols are aggressive and associated with significant short-term toxicity as well as serious long-term health effects for patients who survive [2]. Decades of research and clinical trials have so far failed to improve treatment outcomes for MLL-r leukemia patients [2–5]. Therefore, there is a continued need for the development of novel treatment strategies for MLL-r leukemia, preferentially based on the use of more selective, targeted therapies that are more potent, less toxic and enable the use of lower chemotherapeutic doses for pediatric and infant patients. In this search it is important to consider that MLL-r leukemia is less homogeneous as initially proposed based upon discovery of a common MLL-rearranged leukemia gene-expression signature [6]. Rather, MLL-r leukemia is now thought to be a heterogeneous disease, composed of different disease subtypes, each potentially characterized by a particular gene-expression signature and sustained by subtype-specific disease mechanisms [7–9]. However, more detailed knowledge about these subtypes and associated disease-mechanisms is lacking [7–9].

To identify novel compounds with activity against MLL-r leukemia, we previously developed a cell-based screening method to identify small molecules with selective cytotoxicity against MLL-r leukemia cells [10]. This highly focused discovery strategy previously resulted in the identification of a novel compound, CCI-007, that reverted the aberrant elevated transcription of disease-driving factors *HOXA9*, *MEIS1*, and *CMYC* downstream of the pathogenic MLL fusion protein, thereby inducing MLL-r leukemia cell death [10]. Here, we report the identification of another novel small molecule, CCI-006, that inhibits mitochondrial respiration resulting in insurmountable mitochondrial depolarization and a pro-apoptotic unfolded protein response (UPR) in a subset of MLL-r leukemia cells.

## Results

### Identification of CCI-006 as a novel, selective inhibitor and chemosensitizer of MLL-rearranged leukemia cells

**Fig. 1:**
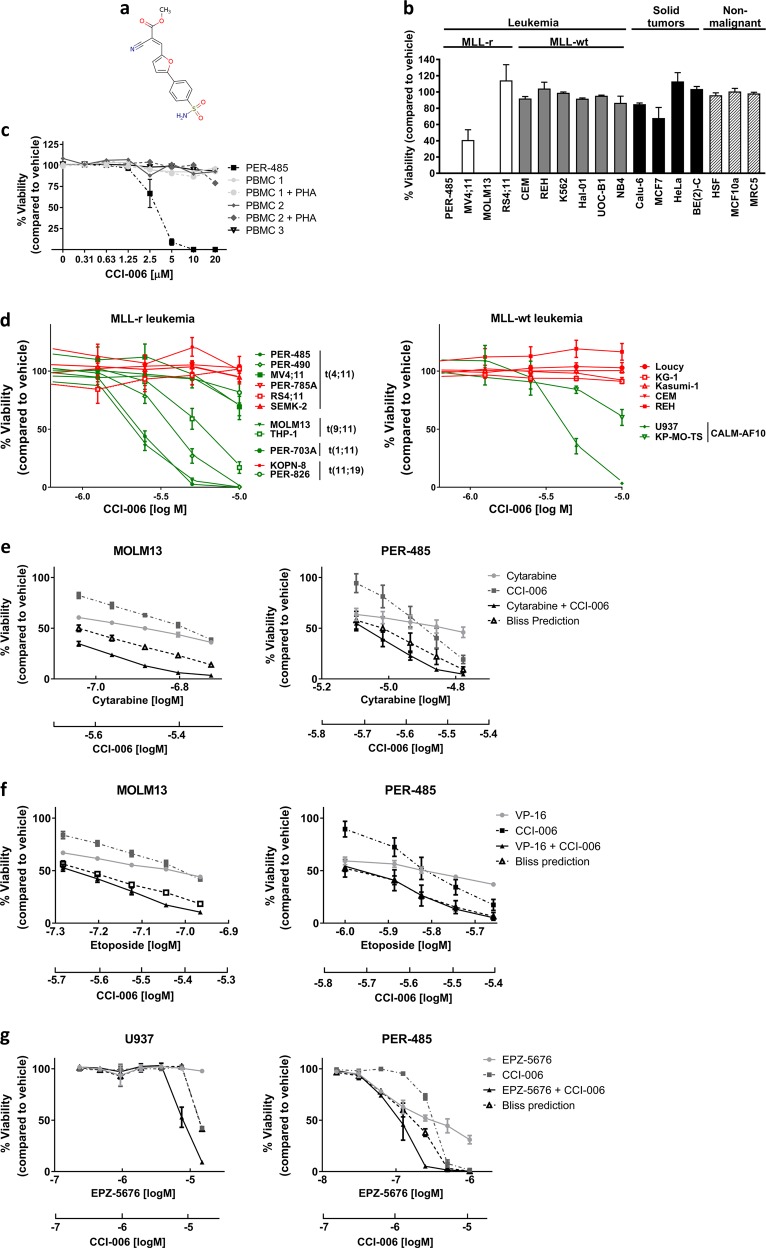
Identification of CCI-006 as a novel selective inhibitor and chemosensitizer of MLL-rearranged leukemia. **a** CCI-006 structure. **b** Cytotoxicity of CCI-006 against a panel of cells as evaluated in resazurin reduction assays. The bar graph shows the percentage viability of a panel of cell lines treated with 10 μM CCI-006 for 72 h relative to vehicle-treated cells. Each data point represents mean ± SEM of at least three independent experiments. **c** Viability of PER-485 cells and peripheral blood mononuclear cells (PBMC) isolated from healthy control donors (*n* = 3) after incubation with a dose range of CCI-006 for 72 h as determined in resazurin reduction assays. PBMCs were tested in the presence and absence of stimulating doses of phytohemagglutinin (PHA). For the PER-485 cells, the viability curve represents mean % viability (relative to vehicle-treated cells) ± SEM of three independent experiments. **d** Cytotoxicity of a dose range of CCI-006 as evaluated by resazurin reduction assays in an expanded leukemia cell line panel. Each data point represents the mean % viability (relative to vehicle-treated cells) ± SEM of at least three independent experiments. **e**–**g** Resazurin reduction synergy assays assessing viability of cells after treatment with CCI-006, cytarabine, etoposide (VP16) or EPZ-5676 as single agents and in combination at indicated doses. Each data point represents mean % viability (relative to vehicle-treated cells) ± SEM of three (cytarabine, VP16) or two (EPZ-5676) independent experiments. The Bliss Prediction curve indicates the predicted % viability of the cells when exposed to the combination of compounds if both compounds work additively together. Synergy is defined as the presence of a lower cell viability percentage upon combination of two compounds compared to the viability predicted based on the presence of an additive effect between the compounds (i.e. the viability curve of the combination runs below the Bliss Prediction curve). **e-f** Cells were treated simultaneously with CCI-006 and chemotherapeutic drug for 72 h. **g** Cells were pre-treated with a dose range of EPZ-5676 for 7 (PER-485) or 10 (U937) days, after which CCI-006 was added for 3 additional days

To identify novel compounds that selectively target MLL-r leukemia, a phenotypic cell-based screen was performed using a chemical small molecule library composed of 34,000 compounds as previously described [10]. The cytotoxic effect of each compound (at 10 μM) was assessed against a leukemia cell line derived from an infant with relapsed MLL-r *t*(4;11) acute lymphoblastic leukemia (ALL), PER-485 [11], by resazurin reduction viability assays. Subsequently, hits were filtered by parallel screens against MLL-wildtype (MLL-wt) cancer cell lines (BE(2)C (neuroblastoma), 22RV (prostate carcinoma), Mel7 (melanoma), and HeLa (adenocarcinoma)). A panel of 30 small molecules was selected that decreased the viability of MLL-r PER-485 cells by more than 70%, while not affecting the viability of any of the MLL-wt cancer cell lines. These 30 hits were further screened against an expanded panel of cell lines, comprising additional MLL-r and MLL-wt leukemia cells, solid tumor cell lines and nonmalignant cells. This screening identified two compounds CCI-006 and CCI-007 that preferentially targeted the tested MLL-r leukemia cell lines (CCI-007 described in [10]). Compound CCI-006 (Fig. 1a) demonstrated a selective cytotoxic effect against three out of four tested MLL-r leukemia cell lines, without significantly affecting the viability of MLL-wt leukemia (*n* = 6) and solid tumor (*n* = 4) cell lines, or noncancerous cells (*n* = 3) at 10 μM (Fig. 1b). Importantly, peripheral blood mononuclear cells from healthy donors (*n* = 3) were not affected by incubation with CCI-006 up to 20 μM (Fig. 1c), providing support for the presence of a therapeutic window for the compound.

To further investigate the observed selective cytotoxicity of CCI-006 towards MLL-r leukemia cell lines, we performed full dose range viability assays with an expanded leukemia cell line panel. We thereby confirmed that CCI-006 selectively decreased the viability of a subset of MLL-r leukemia cells (Fig. 1d, Table 1): the compound reduced the viability of seven out of eleven (64%) “sensitive” MLL-r leukemia cell lines to below 85% of that of vehicle-treated cells at a 10 µM dose, including cell lines derived from infant (younger than 1 year) or pediatric leukemia patients with chemo-resistant disease (Table 1, see Supplementary Table 1 for cell line characteristics) [11]. The remaining four out of eleven (36%) MLL-r leukemia cells were unaffected (>85% viability at 10 µM dose) by CCI-006 treatment and were classified as “unresponsive”. No selectivity of CCI-006 towards a specific *MLL* gene translocation (t(4;11), t(11;19), t(9;11), t(1;11)) or disease type (ALL or acute myeloblastic leukemia) was observed (Table 1). Interestingly, the two out of seven MLL-wt leukemia cell lines that were also sensitive to CCI-006, were characterized by the presence of a CALM-AF10 rearrangement (Fig. 1d). This translocation defines a leukemia subtype closely related to MLL-r leukemia, and is driven by shared mechanisms such as upregulation of the leukemogenic *HOXA9/MEIS1* pathway [12, 13].

**Table 1:** Responsiveness of MLL-r and MLL-wt leukemia cells to CCI-006

Cell line			Translocation	Disease	% Viability^1^	Responsiveness to CCI-006
MLL-r	ALL	PER-485	t(4;11)	Infant ALL	0	Sensitive
		PER-490	t(4;11)	Infant ALL	0	Sensitive
		PER-785A	t(4;11)	Infant ALL	103	Unresponsive
		RS4;11	t(4;11)	Pre-B-cell ALL	114	Unresponsive
		SEMK-2	t(4;11)	Childhood pre-B-cell ALL	100	Unresponsive
		PER-703A	t(1;11)	Infant ALL	70	Sensitive
		PER-826A	t(11;19)	Infant ALL	81	Sensitive
		KOPN-8	t(11;19)	Infant ALL	96	Unresponsive
	AML	MV4;11	t(4;11)	Childhood AML	69	Sensitive
		MOLM13	t(9;11)	AML	0	Sensitive
		THP-1	t(9;11)	Infant AML	17	Sensitive
MLL-wt	ALL	Loucy	SET-NUP214	ALL	103	Unresponsive
		CEM		T-cell ALL	92	Unresponsive
		REH		Pre-B-cell ALL	117	Unresponsive
	AML	U937	CALM-AF10	AML	3	Sensitive
		KP-MO-TS	CALM-AF10	AML	60	Sensitive
		KG-1		AML	91	Unresponsive
		Kasumi-1	AML-ETO	AML	100	Unresponsive

^1^Mean % viability as determined by resazurin reduction assay after 72 h treatment with 10 μM CCI-006, compared to vehicle-treated cells (*n* = 3)

To gain further insight into the clinical potential of CCI-006, we investigated its ability to sensitize MLL-r and CALM-AF10 translocated leukemia cells to chemotherapeutics currently used in the clinic to treat infant and pediatric leukemia patients. Synergy, defined as the presence of a lower cell viability upon combination of two compounds compared to the viability predicted based on the presence of an additive effect of the compounds (Bliss Prediction) [14, 15], was observed between CCI-006 and cytarabine for both MLL-r MOLM13 and PER-485 cell lines (Fig. 1e). Etoposide (VP16) synergized with CCI-006 in the MOLM13 cells, while for the PER-485 cells, an additive effect was observed (Fig. 1f). In addition, we tested whether CCI-006 synergized with the DOT1L inhibitor EPZ-5676 (Pinometostat) that showed modest clinical efficacy in adult patients with advanced acute leukemias, including MLL-r leukemias [5, 13, 16]. Synergy was observed between EPZ-5676 and CCI-006 for the MLL-r PER-485 and CALM-AF10 rearranged U937 cell lines (Fig. 1g).

### CCI-006 induces a pro-apoptotic unfolded protein stress response in sensitive MLL-r leukemia cells within hours of treatment

**Fig. 2:**
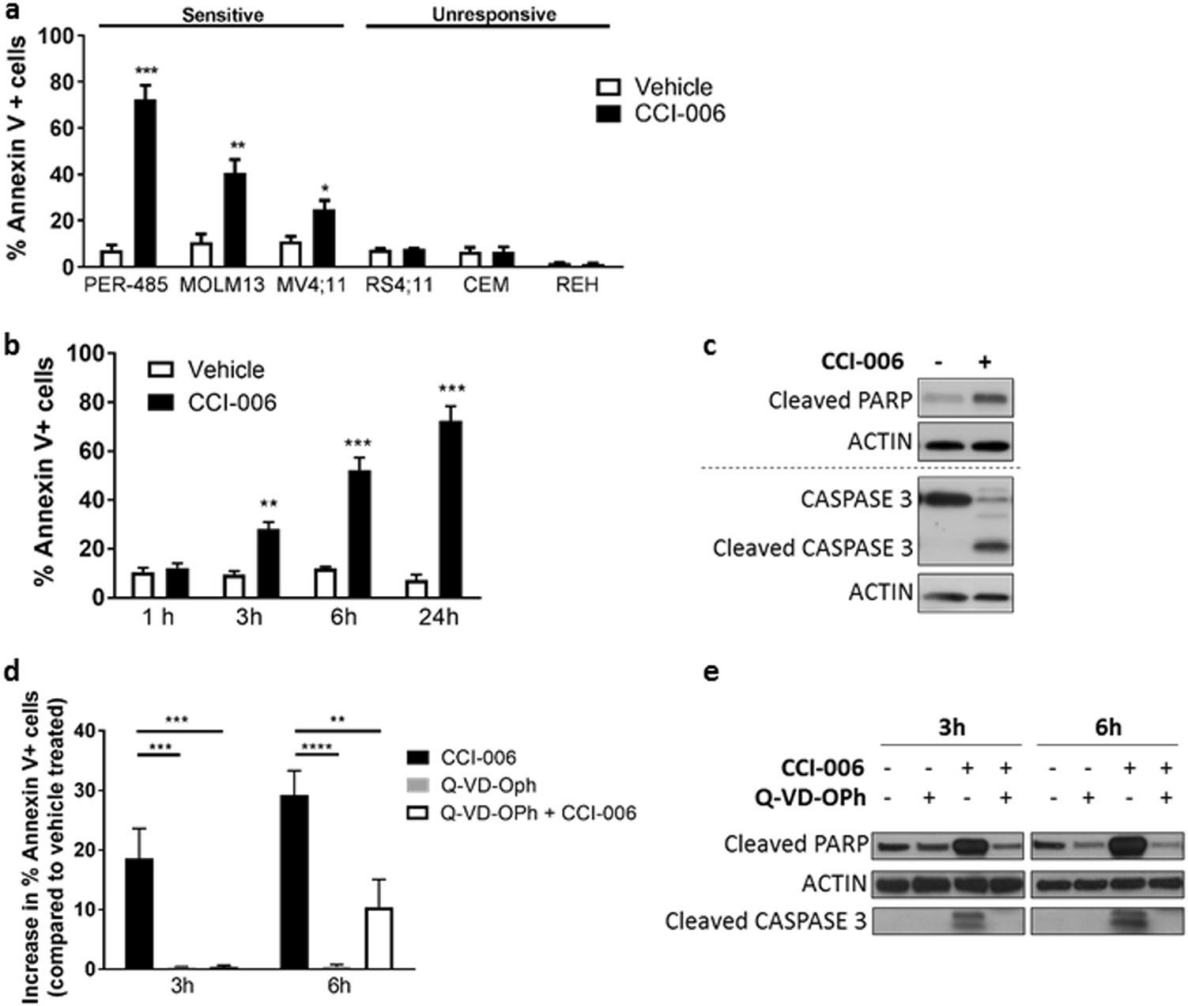
CCI-006 induces apoptosis within a few hours of treatment. **a** CCI-006 induces significant increases in the percentage of Annexin V+ cells in sensitive MLL-r leukemia cell lines PER-485 (*P* = 0.00056), MOLM-13 (*P* = 0.0018) and MV4;11 (*P* = 0.013) compared to vehicle-treated cells, while such an effect was absent in unresponsive CEM, REH, and RS4;11 cells (*P* > 0.05). Bar graph shows mean ± SEM of the percentage of Annexin V+ apoptotic cells as analyzed by flow cytometry following treatment of cells with 5 μM CCI-006 or vehicle for 24 h in at least 3 independent experiments. Mean percentages of Annexin V+ CCI-006-treated and vehicle-treated cells were compared by *t* test. **b** CCI-006 induces a significant increase in the amount of Annexin V+ cells within 3 h treatment of PER-485 cells with 5 μM CCI-006 compared to vehicle-treated cells (*t* test, 3 h: *P* = 0.0044; 6 h: *P* = 0.0002; 24 h: *P* = 0.0006). Bar graph shows mean ± SEM of the percentage of Annexin V+ apoptotic cells as analyzed by flow cytometry following treatment of cells with 5 μM CCI-006 in 3 independent experiments. **c** Western blotting for cleaved PARP and CASPASE 3 using lysates from PER-485 cells treated with 5 μM CCI-006 or vehicle for 24 h. Blot is representative for three independent experiments. **d** Pre-incubation with 10 μM pan-caspase inhibitor Q-VD-OPh for 2 h partly prevents the increase in Annexin V+ cells induced by 5 μM CCI-006 in PER-485 cells. Bar graph depicts mean ± SEM of the increase in percentage of Annexin V+ PER-485 cells following treatment with CCI-006 and Q-VD-OPh either alone or in combination, in comparison to vehicle-treated cells, at various time points as measured by flow cytometry in at least three independent experiments. Mean increases in percentage of Annexin V+ cells between treatment groups were compared by ANOVA followed by Tukey’s multiple comparison test (3 h: *P* = 0.003; 6 h: *P* < 0.0001). **e** Pre-incubation with 10 μM pan-caspase inhibitor Q-VD-OPh for 2 h prevents the induction of PARP and CASPASE 3 cleavage in PER-485 cells treated with 5 μM CCI-006 for 3 and 6 h. Western blot is representative of three independent experiments. *P < 0.05; **P < 0.01; ***P < 0.001; ****P < 0.0001

To understand how CCI-006 decreased the viability of MLL-r leukemia cells, we investigated whether the compound induced apoptosis. CCI-006 significantly increased the percentage of cells expressing cell surface Annexin V (Annexin V+, marker of apoptosis) in sensitive MLL-r leukemia cells, while no apoptosis was observed in unresponsive leukemia cells (Fig. 2a). The increase in the proportion of Annexin V+ PER-485 cells occurred rapidly, within 3 h of treatment (Fig. 2b). Furthermore, treatment with CCI-006 induced the cleavage of PARP and CASPASE 3 (Fig. 2c). Pre-treatment with pan-caspase inhibitor Q-VD-OPh significantly delayed and partially prevented the increase in proportion of Annexin V+ cells (Fig. 2d) as well as completely abolished the cleavage of PARP and CASPASE 3 upon CCI-006 treatment (Fig. 2e), providing evidence that CCI-006 affected MLL-r leukemia cell viability by inducing caspase-mediated apoptosis.

To determine how CCI-006 induced apoptosis in sensitive MLL-r leukemia cells, microarray-based whole-genome expression analysis was performed to identify differentially expressed genes between CCI-006 (5 μM, 3 h) and vehicle-treated MLL-r PER-485 cells. Genego enrichment analysis by Pathway Maps and Process Networks on the list of differentially expressed genes indicated significant enrichment of genes in the Apoptosis—Endoplasmatic Reticulum (ER) stress pathway upon CCI-006 treatment (Table 2). We subsequently confirmed that CCI-006 activated an ER stress response in PER-485 cells: the compound induced a rapid increase in eIF2α phosphorylation (p-eIF2α), exclusively in sensitive MLL-r leukemia cells (Fig. 3a, b), thereby signaling the activation of an unfolded protein response (UPR) that halts cap-mediated mRNA translation to protect against ER stress (reviewed in ref. [17]). This was further confirmed in polysome profiling experiments showing that CCI-006 exposure markedly reduced the amount of actively translating ribosomes in sensitive MLL-r cells, as evidenced by the almost complete abrogation of polysome peaks and a concomitant increase in monosomes (increased 80S peak) in comparison to vehicle-treated cells (Fig. 3c). Although initially protective, a UPR can shift towards engagement of pro-apoptotic pathways upon severe and chronic ER stress, via increased phosphorylation of JNK and increased expression of *CHOP*, as well as through decreasing the levels of short-lived survival proteins by inhibiting translation [18]. CCI-006 treatment increased the levels of *CHOP* mRNA (Fig. 3d) and phosphorylated JNK (p-JNK) (Fig. 3a) and diminished the levels of HOXA9, MEIS1, and CMYC, important leukemogenic drivers and survival factors for MLL-r leukemia (Fig. 3e) in sensitive MLL-r leukemia cells [19–22]. Similar observations were made in sensitive CALM-AF10 translocated leukemia cells treated with CCI-006 (Supplementary Figure 1). The UPR was shown to precede apoptosis as pre-incubation with Q-VD-OPh did not prevent the CCI-006-induced decrease in cellular polysome content (Fig. 3f) and HOXA9 or MEIS1 protein levels (Fig. 3g). In addition, pre-incubation with an inhibitor of PERK (PERKi, GSK2656157), one of the eIF2α-phosphorylating enzymes activated in UPR, significantly and dose-dependently inhibited the increase in Annexin V+ PER-485 cells following CCI-006 treatment (Fig. 3h). This provides evidence that the pro-apoptotic UPR precedes and plays a role in the induction of the mitochondrial, caspase-dependent apoptosis induced by CCI-006.

**Table 2:** Top 5 Pathway Maps and Process Networks identified by Genego enrichment analysis on genes differentially expressed between CCI-006 and vehicle-treated PER-485 cells

#	Process networks/pathway maps	*P* value
*Process networks*		
1	Apoptosis_Endoplasmic reticulum stress pathway	3.81E−03
2	Development_Hedgehog signaling	1.08E−02
3	Development_Ossification and bone remodeling	2.85E−02
4	Apoptosis_Anti-Apoptosis mediated by external signals via MAPK and JAK/STAT	4.31E−02
5	Neurophysiological processes_Corticoliberin signaling	5.09E−02
*Pathway maps*		
1	Development Regulation of CDK5 in CNS	2.13E−04
2	Apoptosis and survival: Endoplasmic reticulum stress response pathway	1.42E−03
3	PGE2 pathways in cancer	1.58E−03
4	Development: Delta- and kappa-type opioid receptors signaling via beta-arrestin	4.18E−03
5	Transcription: Transcription regulation of aminoacid metabolism	4.93E−03

**Fig. 3:**
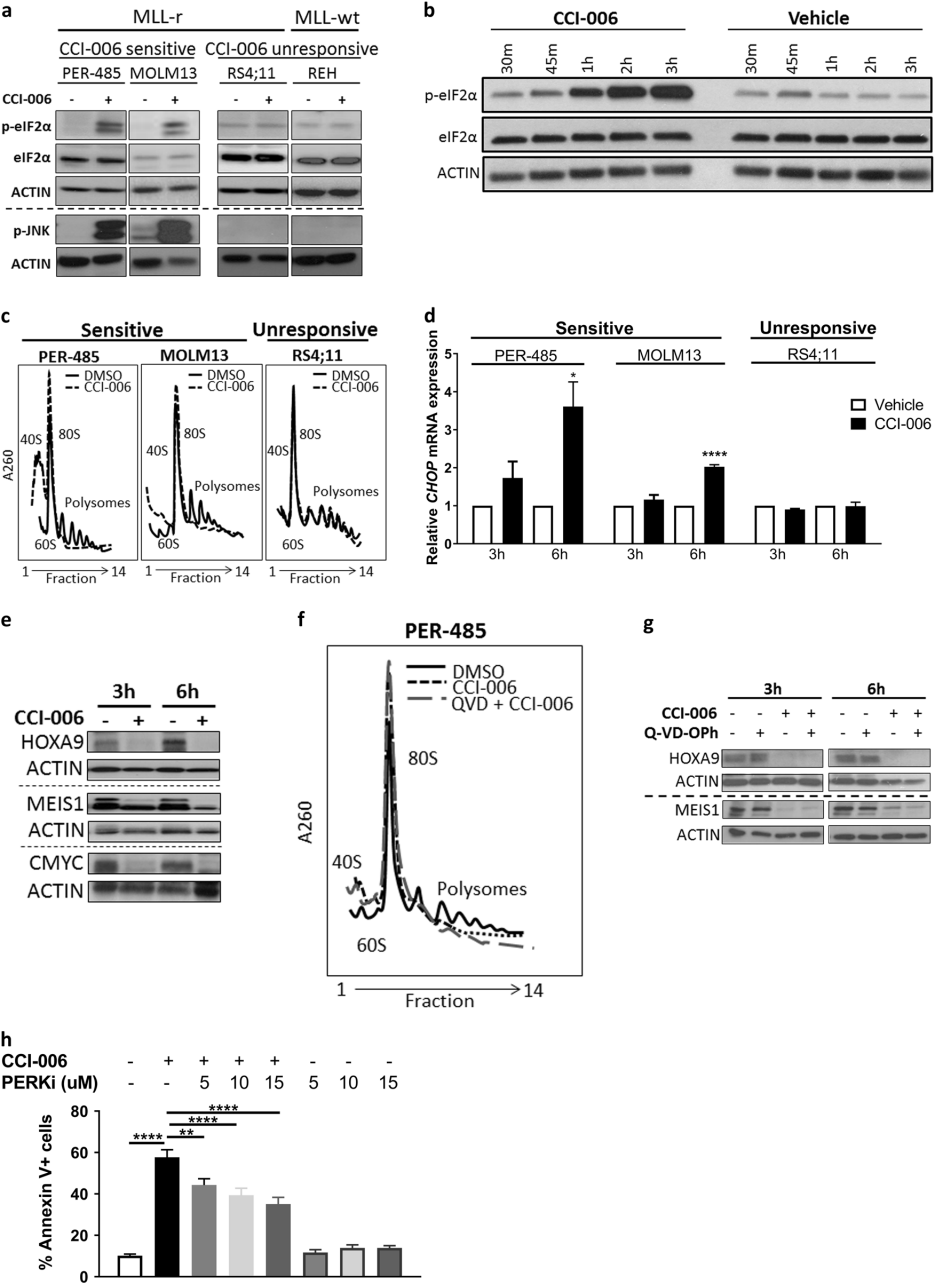
CCI-006 induces a pro-apoptotic unfolded protein response in sensitive MLL-r leukemia cells. **a** Representative Western blot of PER-485 (*n* = 3 independent experiments), MOLM13 (*n* = 3 for eIF2α; *n* = 2 for JNK), RS4;11 (*n* = 3 for eIF2α; *n* = 2 for JNK), and REH (*n* = 3 for eIF2α; *n* = 2 for JNK) cells after a 3h treatment with 5 μM CCI-006 or vehicle. **b** Representative immunoblotting of PER-485 cells treated with 5 μM CCI-006 or vehicle for 30 min (30 m) up to 3 h (2 independent experiments). **c** Representative result of a polysome profiling experiment performed on PER-485 (*n* = 3 independent experiments), MOLM13 (*n* = 3) and RS4;11 (*n* = 2) cells treated with 5 μM CCI-006 or vehicle for 3 h. **d** *CHOP* mRNA levels were assayed in cells treated with 5 μM CCI-006 or vehicle for 3 h by quantitative RT-PCR and relative expressions were calculated using the ΔΔCt method. Gene expressions were normalized against housekeeping genes and expressed relative to vehicle-treated cells. Assays were run in duplicate within each experiment. Each data point represents the mean ± SEM of three independent experiments. Mean relative expressions were compared between CCI-006 and vehicle-treated cells groups by *t* tests. **e** Representative Western blot of PER-485 cells treated with 5 μM CCI-006 or vehicle for 3 and 6 h (*n* = 2 independent experiments). **f** Pre-incubation with 10 μM Q-VD-OPh for 2 h did not affect the CCI-006-induced effect on the polysome profile of PER-485 cells. Representative result of two independently performed polysomal profiling experiments. **g** Pre-incubation with 10 μM pan-caspase inhibitor Q-VD-Oph for 2h prevents the CCI-006-induced decrease in protein levels of HOXA9 and MEIS1. Western blot is representative of three independent experiments. **h** Bar graph depicts mean ± SEM of the percentage of Annexin V+ PER-485 cells following pre-treatment with different dosages of PERK inhibitor GSK2656157 (or vehicle) followed by incubation with 5 μM CCI-006 for 6 h (or vehicle) as measured by flow cytometry in at least three independent experiments. Mean increases in percentage of Annexin V+ cells were compared by ANOVA (*P* < 0.0001) followed by Tukey’s multiple comparison test: *P < 0.05; **P < 0.01; ****P < 0.0001

### Resistance of MLL-r leukemia cells to CCI-006 is associated with a glycolytic metabolic phenotype

**Fig. 4:**
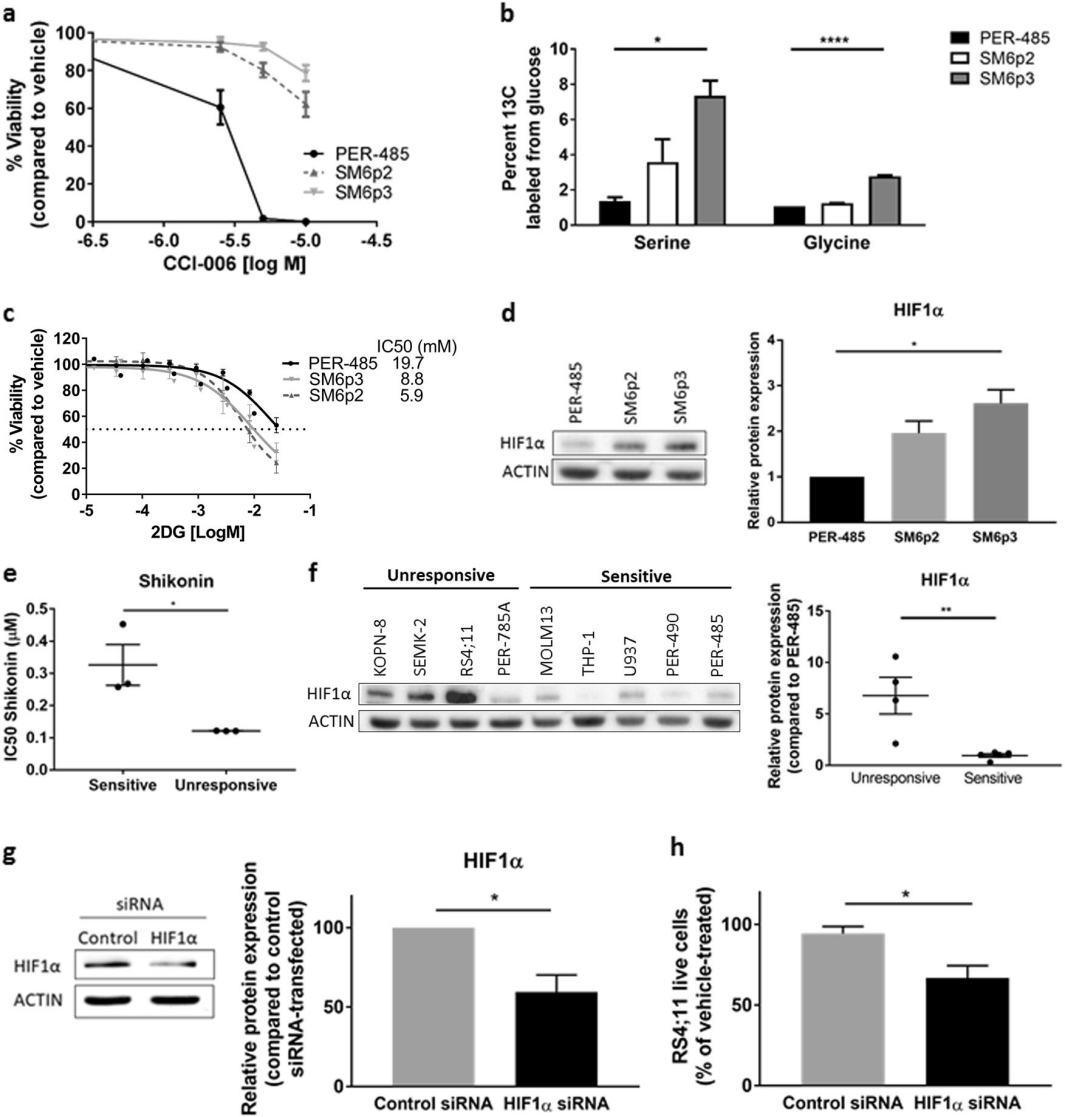
Resistance of MLL-r leukemia cells to CCI-006 is associated with a glycolytic metabolic phenotype. **a** Viability of PER-485, SM6p2, and SM6p3 cells upon incubation with a dose range of CCI-006 for 72 h as evaluated by resazurin reduction assays. Each data point represents mean % viability (relative to vehicle-treated cells) ± SEM of at least three independent experiments. **b** Metabolite labeling of PER-485, SM6p2, and SM6p3 with ^13^C glucose for 24 h followed by mass spec-based quantitation of labeled metabolite species. Samples were run in duplicate within each experiment. Bar graph represents the mean percentage of ^13^C incorporation ± SEM of two independent experiments. Mean ^13^C incorporation in cell lines was compared by ANOVA followed by Tukey’s multiple comparison test. **c** Viability of PER-485, SM6p2, and SM6p3 upon incubation with a dose range of 2DG for 72 h as evaluated in a resazurin reduction assays. Each data point represents mean % viability (relative to vehicle-treated cells) ± SEM of at least two independent experiments. **d** Representative immunoblot for baseline HIF1α levels in PER-485, SM6p2, and SM6p3 cells (*n* = 2 independent experiments) and quantification of protein expression relative to ACTIN and normalized to PER-485 cells. Mean relative protein expressions between cell lines were compared by ANOVA followed by Tukey’s multiple comparison test. **e** IC50s for shikonin as determined by resazurin reduction viability assays on a panel of CCI-006-sensitive (PER-485, MOLM13, and U937) and unresponsive (RS4;11, KOPN-8, and SEMK-2) leukemia cells. Each point represents the mean IC50 of a cell line as determined in at least two independent experiments. Mean IC50s per group were compared by *t* test. **f** Representative blot for HIF1α in a panel of unresponsive and sensitive MLL-r and CALM-AF10 leukemia cells. Densitometry was performed on blots from two independent experiments and protein expression was normalized to ACTIN, followed by normalization across cell lines to PER-485 cells. Relative HIF1α protein expressions in sensitive and unresponsive cells were compared by *t* test. **g** RS4;11 cells were transfected with nonviral star nanoparticle-siRNA (control and HIF1α) for 18 h and a representative immunoblotting for the protein level of HIF1α is shown. Protein expression was quantified by densitometry and normalized to ACTIN, followed by normalization of the relative expression level of HIF1α to the level in control siRNA-transfected cells. Bar graphs represent the mean relative protein expression ± SEM in three independently performed transfection experiments. The significance of the decrease in relative HIF1α expression in HIF1α siRNA-transfected cells was evaluated by one sample *t* test. **h** RS4;11 cells were transfected with star nanoparticle-siRNA (control and HIF1α) complexes for 18 h followed by treatment with CCI-006 (12.5 µM, 6 h) or vehicle. Bar graphs represents the mean % of viable cells after CCI-006 treatment relative to vehicle-treated cells ± SEM as determined by Trypan blue exclusion assay. Mean % of viable cells between control siRNA and HIF1α siRNA-transfected cells were compared by *t* test. *P < 0.05; **P < 0.01; ****P < 0.0001

Numerous physiological and non-physiological cellular processes can culminate in the activation of a pro-apoptotic UPR [17]. To obtain more insight into why CCI-006 induced a pro-apoptotic UPR in a subset of MLL-r leukemia cells while the unresponsive MLL-r leukemia cells were unaffected, we generated resistant pools from the inherently sensitive MLL-r leukemia PER-485 cells, by subjecting this cell line to multiple rounds of selection in CCI-006 (Fig. 4a). The reduced responsiveness of the two resulting pools SM6p2 and SM6p3 to CCI-006 was similar in nature to that of the intrinsically unresponsive MLL-r leukemia cells, as exemplified by the absence of apoptosis induction, stable protein levels of MLL gene targets HOXA9, MEIS1, and CMYC, unchanged polysomal profiles and unchanged levels of p-eIF2α and p-JNK upon treatment with CCI-006 (Supplementary Figure 2a–d).

To investigate the underlying mechanism of acquired resistance to CCI-006 in SM6p2 and SM6p3 cells, we performed microarray gene expression analysis on these cells and the parental PER-485 cells. Gene annotation enrichment analysis (DAVID) on genes differentially expressed between the resistant pools and PER-485 cells (fold change (FC) > |2| or *P* < 0.05) showed no significant enrichments for the SM6p2 cells, while 7 of the 13 enriched KEGG pathways in SM6p3 cells were related to cellular metabolism (Table 3). Differentially expressed genes encoded for proteins involved in energy metabolism, anabolism and catabolism, suggesting that the SM6p3 cells reprogrammed their metabolism (Supplementary Table 2). Analysis of the individual differentially expressed genes indicated an upregulation of key enzymes in the serine biosynthesis pathway in SM6p3 (phosphoserine phosphatase, FC = 2.05; phosphoglycerate dehydrogenase, FC = 5.06) and SM6p2 cells (phosphoglycerate dehydrogenase, FC = 2.62), suggesting that SM6p2 and SM6p3 cells had undergone changes in their serine/glycine biosynthesis pathways (Supplementary Figure 2e). Gene set enrichment analysis provided further evidence that SM6p3 cells had acquired alterations in their gene-expression profile related to mitochondrial respiration and HIF1α-regulated cellular metabolism (Supplementary Figure 3a–c).

**Table 3:** Gene annotation enrichment analysis on genes differentially expressed between parental PER-485 and SM6p3 cells by DAVID

#	KEGG pathway	Number of genes^1^	*P* value
1	Biosynthesis of antibiotics	22	1.20E−06
2	Ribosome	15	5.10E−05
3	Carbon metabolism	12	5.30E−04
4	Metabolic pathways	50	1.80E−02
5	Biosynthesis of amino acids	7	2.30E−02
6	Glycolysis / Gluconeogenesis	6	5.00E−02
7	Pentose phosphate pathway	4	5.50E−02
8	Epstein–Barr virus infection	11	5.90E−02
9	Lysosome	8	7.10E−02
10	Cell cycle	8	7.80E−02
11	Pathways in cancer	18	8.00E−02
12	Alanine, aspartate and glutamate metabolism	4	8.60E−02
13	Dopaminergic synapse	8	8.90E−02

^1^Genes described in detail in Supplementary Table 2

To confirm that SM6p2 and SM6p3 cells metabolically reprogrammed in response to CCI-006 exposure, ^13^C-metabolite labeling studies were performed. Greater labeling of both serine and glycine was observed in SM6p3 compared to the parental PER-485 line, indicating greater de novo synthesis of these metabolites in the resistant SM6p3 cell line, while a trend was seen for the SM6p2 cells (Fig. 4b). In addition, SM6p2 and SM6p3 were more sensitive to glycolysis inhibitor 2-deoxyglucose (2DG) compared to the parental PER-485 cells (Fig. 4c). Moreover, SM6p2 and SM6p3 had increased expression levels of HIF1α, a transcriptional regulator of hypoxic response that has been shown to mediate the cellular metabolic switch from mitochondrial metabolism toward glycolysis (Fig. 4d). Together, these results point toward the induction of an altered, more glycolytic, metabolic profile in the SM6p2 and SM6p3 cell pools compared to the parental cells, suggesting that these cells were forced to metabolically reprogram to acquire resistance to continuous CCI-006 treatment.

We next investigated whether this mechanism of acquired resistance was representative for resistance by the intrinsically unresponsive MLL-r leukemia cells. Similarly to the cells with acquired resistance to CCI-006, the MLL-r leukemia cells with intrinsic resistance were significantly less responsive to glycolysis inhibitor shikonin than the sensitive MLL-r leukemia cells, while a similar trend was observed for 2DG (Fig. 4e, Supplementary Figure 2f, g). Moreover, in analogy to SM6p2 and SM6p3, the intrinsically unresponsive cells expressed significantly higher baseline levels of HIF1α compared to the sensitive MLL-r leukemia cells, confirming that intrinsically unresponsive MLL-r leukemia cells inherently present with a more glycolytic phenotype than the responsive cells (Fig. 4f).

To further determine the functional significance of this glycolytic phenotype and the HIF1α pathway in MLL-r leukemia cell line resistance to CCI-006, we investigated the effect of *HIF1A* gene silencing on the responsiveness of the resistant MLL-r RS4;11 cell line to CCI-006. To deliver HIF1A siRNA into MLL-r leukemia cells we used a nonviral lipid nanoparticle developed by our group to self-assemble and deliver siRNA to tumor cells [23]. Transfection of HIF1A siRNA significantly decreased HIF1α levels in the unresponsive RS4;11 cells (Fig. 4g) and sensitized the cell line toward CCI-006 while cells transfected with control siRNA remained unaffected (Fig. 4h). These findings add significant strength to our hypothesis that the observed intrinsic resistance of MLL-r leukemia cells to CCI-006 is mediated through the HIF1α pathway.

### CCI-006 induces mitochondrial dysfunction in sensitive MLL-r leukemia cells

**Fig. 5:**
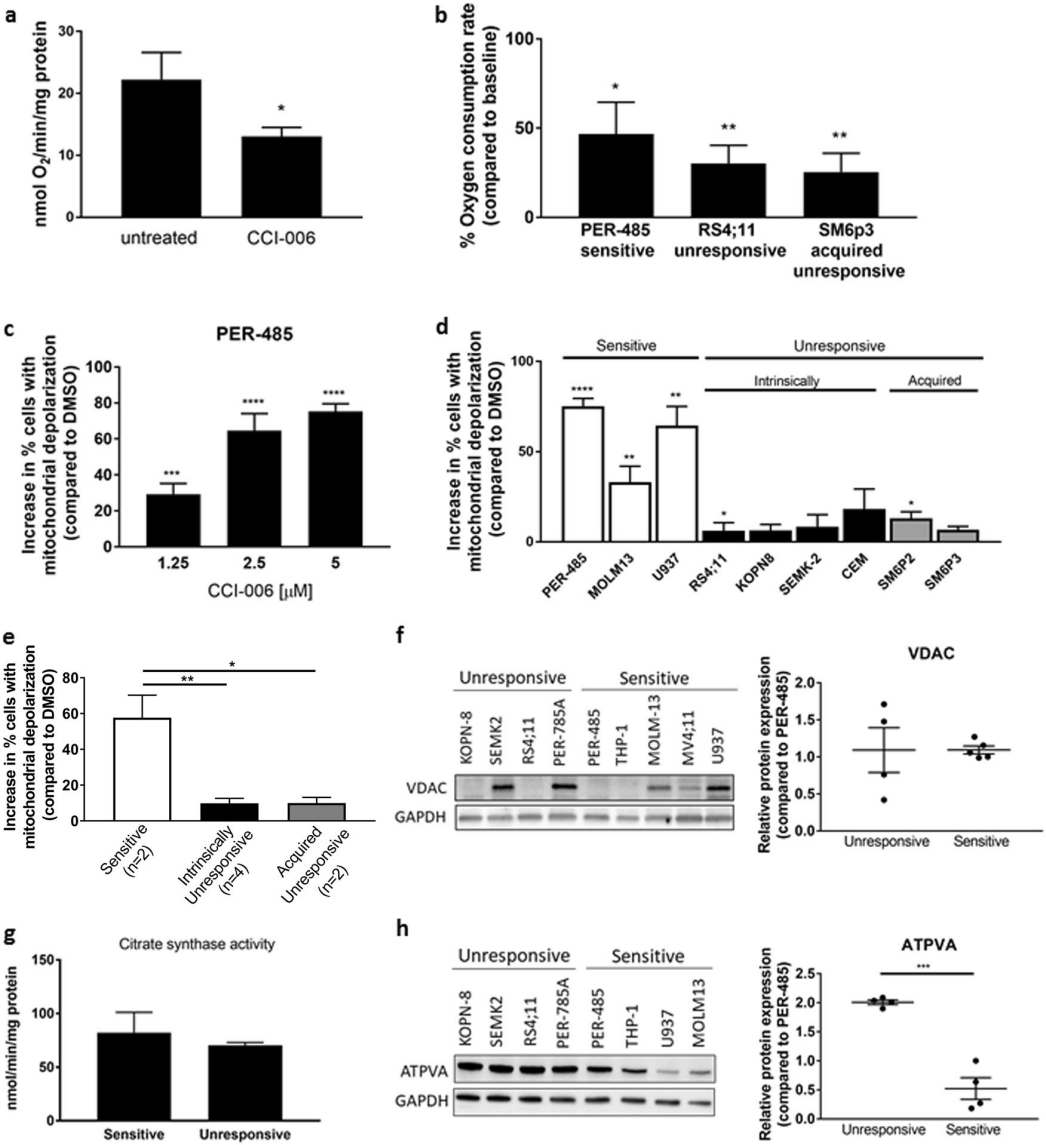
CCI-006 affects mitochondrial functioning in sensitive MLL-r leukemia cells. **a** Oxygen consumption rate in mouse liver mitochondria, untreated and treated with 1 μM CCI-006 as measured by Clark electrode. Bar graphs represent the mean oxygen consumption ± SEM in mitochondrial preparations from five mice. Mean oxygen consumption after treatment was compared to untreated, baseline OCR by paired *t* test. **b** Percentage oxygen consumption rate in cell lines treated with 1 μM CCI-006 compared to baseline OCR in untreated cells. Bar graphs represent the mean percentage OCR of treated cells compared to baseline ± SEM of measurements performed over two independent days. Mean percentages OCR in treated cell lines were compared to baseline OCR by ANOVA. **c**, **d** Increase in the percentage of cells with depolarized mitochondria after 1 h CCI-006 treatment with a dose response (**c**) or 5 μM (**d**) CCI-006 compared to vehicle-treated cells as determined based on JC-1 staining. Bar graphs represent the mean of three independent experiments ± SEM. *T* tests were used to compare the mean percentage of cells with depolarized mitochondria in CCI-006 vs. vehicle-treated cells. **e** Comparison of mean percentages of cells with depolarized mitochondria ± SEM after 1 h of 5 μM CCI-006 treatment of (PER-485, MOLM13, and U937), intrinsically unresponsive (RS4;11, KOPN8, SEMK-2, and CEM) and acquired unresponsive (SM6p2 and SM6p3) cells by ANOVA followed by Tukey’s multiple comparison. **f** Representative blot for VDAC in a panel of unresponsive and sensitive MLL-r and CALM-AF10 leukemia cells. Densitometry was performed on blots performed on at least two independent cell harvests and protein expressions were normalized to GAPDH, followed by normalization across cell lines to PER-485 cells. Each dot point represents the mean relative protein expression of VDAC in one cell line. Relative protein expressions in sensitive and unresponsive cells were compared by *t* test. **g** Mean baseline citrate synthase activity in sensitive (*n* = 3) and unresponsive (*n* = 4) MLL-r and CALM-AF10 leukemia cells ± SEM. Mean citrate synthase activities in sensitive and unresponsive cells were compared by *t* test. **h** To investigate the composition of the mitochondrial respiratory chain, immunoblotting was performed for protein subunits of the respiratory complex that are labile when not assembled in a respiratory complex. The level of each protein in this immunoblot is thus representative for the relative amount of the respective respiratory chain complex to which it belongs. Representative immunoblot for ATP5A in a panel of unresponsive and sensitive MLL-r and CALM-AF10 leukemia cells. Densitometry was performed on blots from at least two independent experiments and protein expressions were normalized to GAPDH, followed by normalization across cell lines to PER-485 cells. Each dot point represents the mean relative protein expression of ATP5A in one cell line. Relative ATP5A protein expression in sensitive and unresponsive cells was compared by *t* test. *P < 0.05; **P < 0.01; ***P < 0.001; ****P < 0.0001

The fact that acquired and intrinsic resistance to CCI-006 was associated with a more glycolytic metabolic profile suggests that CCI-006 might induce a pro-apoptotic UPR through affecting metabolism. Based on several studies reporting that cells exposed to mitochondrial stressors undergo similar transcriptional and phenotypic changes as SM6p2 and SM6p3, we hypothesized that CCI-006 might affect mitochondrial functioning [24–26]. To examine the direct effects of CCI-006 on mitochondrial function, oxygen consumption rate (OCR) and mitochondrial respiration were measured in isolated mouse liver mitochondria following treatment with the compound. CCI-006 effectively and significantly decreased basal respiration in mouse liver mitochondria as measured by a Clark-type electrode (Fig. 5a), while a trend was noted for an attenuation of ADP-stimulated respiration (Supplementary Figure 4a). We subsequently confirmed that CCI-006 had a similar effect on leukemia cell lines; CCI-006 significantly decreased OCR in all tested cell lines (PER-485, RS4;11, and SM6p3), irrespective of the sensitivity of the cell toward CCI-006 (Fig. 5b). In addition, in a JC-1 assay for mitochondrial membrane depolarization, CCI-006 induced mitochondrial depolarization in a dose-dependent manner within 1 h of treatment (Fig. 5c). However, despite the fact that CCI-006 reduced mitochondrial respiration in both sensitive and unresponsive cells, the increase in percentage of depolarized cells was significantly greater in sensitive MLL-r (PER-485, MOLM13) and CALM-AF10 (U937) cells (average 58% across cell lines), compared to the unresponsive leukemia cells (averaging 10%) (Fig. 5d, e, Supplementary Figure 4b). The extent of depolarization in the unresponsive cells did also not increase upon longer incubation with the compound (Supplementary Figure 4c), indicating that the unresponsive cells did not undergo mitochondrial membrane depolarization upon CCI-006 addition. As the mitochondrial membrane depolarization was observed in the responsive cells within one hour of treatment, this effect preceded, and was not secondary to CCI-006 induced apoptosis (AnnexinV increase after 3 h, Fig. 2b).

These findings suggest that sensitive and unresponsive cells react differently to the CCI-006-induced decrease in OCR, with sensitive cells undergoing mitochondrial membrane depolarization, UPR and apoptosis while the unresponsive cells remain unaffected. To determine whether this differential response of sensitive vs. unresponsive MLL-r leukemia cells was mediated through baseline differences in mitochondrial content and activity, we investigated baseline citrate synthase activity and VDAC protein levels as markers for mitochondrial content and activity. No significant differences were observed in baseline VDAC levels (Fig. 5f) or citrate synthase activity (Fig. 5g) between sensitive and unresponsive MLL-r leukemia cells, indicating that these cells do not vary in baseline mitochondrial content and activity. However, immunoblotting for protein subunits of respiratory complexes showed that the level of Mitochondrial complex V protein ATP5A (complex V ATP synthase) was significantly lower in the sensitive MLL-r leukemia cells (Fig. 5h) while levels of respiratory complex II and IV proteins (SDHB and COX2, respectively) did not differ between sensitive and unresponsive cells (Supplementary Figure 5a). As the observed level of each respiratory complex protein is representative for the amount of respiratory complex to which it belongs, this suggests that the sensitive cells have less assembled Mitochondrial complex V ATP synthase and thus a differentially constituted respiratory chain compared to the unresponsive cells. The difference in mitochondrial respiratory chain constitution between sensitive and unresponsive MLL-r cells was however not associated with a general increased sensitivity to other mitochondrial stressors carbonyl cyanide *m*-chlorophenyl hydrazone (CCCP), a mitochondrial uncoupler, or oligomycin, a mitochondrial complex V inhibitor (Supplementary Figure 5b).

### MLL-r leukemia cells sensitive to CCI-006 express lower levels of MEIS1 than CCI-006-resistant MLL-r leukemia cells

**Fig. 6:**
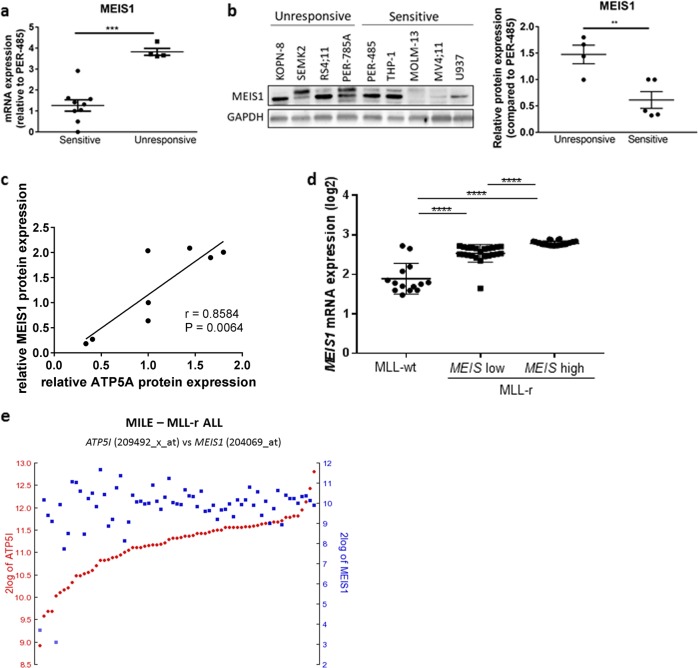
MLL-r leukemia cells sensitive to CCI-006 express lower levels of MEIS1 than CCI-006-resistant MLL-r leukemia cells. **a** To assess baseline expression of genes and proteins of interest, cell pellets were harvested from cells in exponential growth, three times independently for each cell line. *MEIS1* mRNA levels were assayed by quantitative RT-PCR in a panel of sensitive and unresponsive MLL-r and CALM-AF10 cell lines. Relative gene expression was calculated using the ΔΔCt method. Gene expression was normalized against housekeeping genes and expressed relative to PER-485 baseline expression levels. Assays were run in triplicate within each experiment and each data point in the graph represents the mean ± SEM of at least three independent experiments. Group means were compared by *t* test. **b** Representative immunoblotting for MEIS1 using lysates from leukemia cells harvested in exponential growth. The graph depicts quantification data from three independently performed experiments. Protein levels of MEIS1 were quantified relative to the housekeeping protein GAPDH and normalized to the value obtained for PER-485 cells. Group means of relative expression were compared by *t* test. **c** Correlation between relative MEIS1 and ATP5A protein levels in a panel of unresponsive and sensitive MLL-r and CALM-AF10 leukemia cells (Pearson). **d** Analysis of *MEIS1* mRNA expression levels in the Stam et al. [8] pediatric ALL database. MLL-r patients were divided in “*MEIS* low” and “*MEIS* high” based on a cut-off defined by the highest *MEIS1* mRNA expression measured in the MLL-wt cohort. **e** Messenger RNA expression levels of *MEIS1* and *ATP5I* in the MILE database (*n* = 67 MLL-r leukemia patients) as analyzed through R2. **P < 0.01; ***P < 0.001; ****P < 0.0001

MLL-r leukemia cells sensitive to CCI-006 thus present with a different metabolic profile compared to unresponsive MLL-r leukemia cells, exemplified by lower expression levels of HIF1α, a greater resistance to glycolysis inhibitors and a differentially composed mitochondrial respiratory chain. These results are particularly interesting considering the recently proposed role of the MLL target gene *MEIS1*, in the regulation of metabolic phenotype and oxidative phosphorylation in leukemia cells and stem cells [27, 28] and the identification of *HIF1A* as a target gene of MEIS1 [27]. Upon assessing expression levels of MEIS1 in a panel of CCI-006-sensitive and unresponsive cells, the unresponsive MLL-r leukemia cells expressed significantly higher mRNA and protein levels of MEIS1 compared to the sensitive cells (Fig. 6a, b). In addition, although no correlation was observed between MEIS1 and HIF1α protein levels (data not shown), a positive correlation was noted between the protein expression levels of MEIS1 and ATP5A in the leukemia cell line panel (Fig. 6c) [19–22].

To investigate whether this variability in levels of MEIS1 expression is observed within MLL-r patient samples, the mRNA expression of *MEIS1* was investigated in a large pediatric ALL patient dataset (MLL-wt: *n* = 14; MLL-r: *n* = 59) [8]. We found that, although the MLL-r leukemia patient group as a whole presents with significantly higher average levels of *MEIS1* mRNA compared to MLL-wt leukemia patients, *MEIS1* mRNA expression levels vary within the MLL-r leukemia patient subset, and for some patients are within the ranges found for MLL-wt leukemia patients, reflecting our findings in MLL-r leukemia cell lines (Fig. 6d). Although *MEIS1* mRNA levels did not correlate with *HIF1A* expression (data not shown), a positive correlation was noted between *MEIS1* expression levels and mRNA levels of the mitochondrial Complex V ATP synthase subunit (*ATP5I*) in a cohort of MLL-r leukemia patients (R2, https://r2.amc.nl) (*n* = 67, Pearson’s *r* = 0.427, *P* < 0.001, Fig. 6e). A similar trend for a correlation was observed in a smaller MLL-r leukemia patient cohort (*n* = 21, Supplementary Figure 6), indicating a possible link between *MEIS1* and mitochondrial respiratory chain composition.

### CCI-006 and CCI-007 target a similar subset of MLL-r leukemia cells but exert their cell-killing effects differently

As previously reported, our screening approach for inhibitors of MLL-r leukemia resulted in the identification of another novel compound, CCI-007, that demonstrated cytotoxic activity against a subset of MLL-r and CALM-AF10 translocated leukemia cell lines [10]. Interestingly, we observed that both CCI-006 and CCI-007 largely target the same subset of leukemia cell lines, with exception of the SET-NUP214 translocated Loucy cell line which is sensitive to CCI-007 but not to CCI-006 treatment. However, CCI-006 and CCI-007 are structurally unrelated (Fig. 1a) [10]. In addition, each compound has a different mechanism of killing MLL-r leukemia cell lines. We previously showed that CCI-007 affects the survival of MLL-r leukemia cells by reverting the disease-driving MLL-r leukemia gene-expression signature and reducing the mRNA expression levels of *HOXA9*, *MEIS1*, *CMYC*, and *BCL2*, followed by a decrease in protein levels of these important disease drivers within three hours of exposure [10]. In contrast to CCI-007-treated PER-485 cells [10], global gene-expression analysis of CCI-006-treated PER-485 cells did not reveal a reversal of the MLL-r leukemia target gene signature upon CCI-006 treatment (Table 2). This was directly confirmed by quantitative RT-PCR, whereby we demonstrated that CCI-006, unlike CCI-007, did not decrease the mRNA expression of leukemogenic MLL target genes *HOXA9* and *MEIS1* in MLL-r leukemia cells (Supplementary figure 7a). Also, in concordance with their distinct mechanisms of action, CCI-006 and CCI-007 are characterized by different kinetics of cell killing. In the MLL-r PER-485 cells, CCI-006 induces mitochondrial membrane depolarization within 1 h of treatment, prior to the induction of apoptosis (significant increase in apoptotic cell number within 3 h) (Fig. 5c–e, Fig. 2b). CCI-007 on the other hand, requires a longer time frame (between 6 and 24 h) to significantly increase the extent of mitochondrial membrane depolarization (Supplementary Figure 7b, c) and proportion of apoptotic cells [10].

**Fig. 7:**
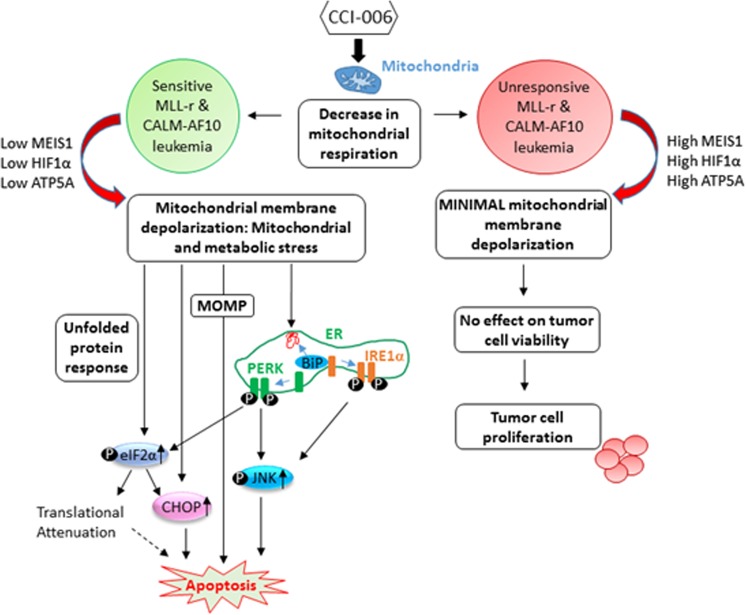
Model of CCI-006 mechanism of action. CCI-006 inhibits cellular mitochondrial respiration. In sensitive MLL-r and CALM-AF10 leukemia cells, characterized by low levels of MEIS1, HIF1α, and ATP5A, mitochondrial depolarization ensues. The compound thereby induces mitochondrial and metabolic stress, activating an unfolded protein response. Mitochondrial dysfunction affects cellular metabolic and redox balance, disturbances of which are described to have an effect on ER homeostasis, resulting in protein misfolding and the attraction of BiP as a chaperone to repair protein folding, culminating in the release and activation of PERK and IRE1α. Upon ER stress, PERK phosphorylates eIF2α which blocks global cap-mediated translation. The phosphorylation of eIF2α also results in an ATF4-mediated increase in expression of the pro-apoptotic CHOP. Similarly, IRE1α activation can induce increased phosphorylation of JNK which has been shown to induce apoptosis. Mitochondrial stress by itself can contribute to a cellular unfolded protein response that is characterized by increased expression of pro-apoptotic CHOP and p-JNK. Furthermore, mitochondrial dysfunction and loss of mitochondrial membrane potential can ultimately result in mitochondrial outer membrane permeabilization (MOMP), a well-described process in the mitochondria-mediated apoptosis pathway. All these pathways culminate in apoptosis induction in sensitive MLL-r and CALM-AF10 leukemia cells. No mitochondrial membrane depolarization is observed in MLL-r and CALM-AF10 leukemia cells with high MEIS1, HIF1α, and ATP5A upon incubation with CCI-006, and the viability of these cells remains unaffected by the compound

These data are in line with and further support the unique mechanism of action by which CCI-006 induces apoptosis in sensitive MLL-r leukemia cells, namely by rapidly inhibiting mitochondrial respiration, thereby inducing mitochondrial membrane depolarization, an UPR and apoptosis in a subset of MLL-r and CALM-AF10 leukemia cells characterized by relatively lower expression levels of MEIS1 and HIF1α (Fig. 7).

## Discussion

Despite the fact that MLL-rearranged leukemia is characterized by the presence of a common gene-expression signature [6], the disease is now considered to be heterogeneous and composed of several disease subtypes defined by specific gene-expression signatures and subtype-specific leukemogenic mechanisms [7–9]. However, knowledge about these subtypes and their underlying pathways is practically nonexistent and this has limited the development of tailored therapeutic strategies to target those specific leukemogenic pathways that drive disease in patient subgroups [9].

Our search for novel compounds that selectively affect the survival of MLL-r leukemia cells yielded CCI-006 and CCI-007 that largely target an identical subset of MLL-r and CALM-AF10 translocated leukemia cell lines. Despite their clearly distinct structure and mechanism of action, it is possible that CCI-007 and CCI-006 both affect the same subset of MLL-r leukemia cells by targeting different underlying sensitivities or characteristics associated with this subset. We showed that the group of sensitive MLL-r leukemia cells is characterized by a particular gene expression profile (low *HOXA9*, *MEIS1*, and *CMYC* mRNA levels) [10] and low levels of MEIS1/HIF1α protein expression. CCI-007 seems to target and affect the characteristic MLL target gene signature of this subset of cells, decreasing the mRNA expression levels of known MLL-r leukemia drivers. At the same time, this subset of cells could be sensitive to the mitochondrial respiration inhibition induced by CCI-006 through an underlying metabolic vulnerability downstream of this characteristic MLL target gene-expression signature mediated by low MEIS1/HIF1α levels.

Our study on CCI-006 thus revealed a dichotomous susceptibility of MLL-r leukemia cells to respiration inhibition by CCI-006 and provided thereby evidence for the existence of metabolic variability within MLL-r leukemia. Insight into this inherent metabolic variability was obtained by analyzing changes present in MLL-r leukemia cells with induced, acquired resistance to CCI-006. We showed that the MLL-r leukemia cells with acquired resistance to the compound underwent genetic and phenotypic changes (increased sensitivity to glycolysis inhibitors, increased HIF1α levels) corresponding to reprogramming toward a more glycolytic metabolic profile compared to the parental cells from which they were derived. We subsequently confirmed that the characteristics of induced resistance were also present in MLL-r leukemia cells intrinsically resistant to CCI-006; similarly to the cells with acquired resistance, the intrinsically resistant cells were more sensitive to glycolysis inhibitors and presented with elevated HIF1α levels compared to intrinsically sensitive MLL-r leukemia cells.

The intrinsically resistant MLL-r leukemia cells also presented with elevated MEIS1 expression compared to the sensitive cells. The association between CCI-006 sensitivity and baseline expression levels of MEIS1 is interesting in light of increasing evidence for an important role of *MEIS1* in leukemogenesis. *MEIS1* has historically been described as a *HOX* cofactor, collaborating with *HOX* genes in the development of leukemia [19, 29]. However, several more recent studies indicate that *MEIS1* alone might be a more important leukemogenic factor than *HOXA9* [28, 30]. This is corroborated by a recent study in pediatric *t*(4;11) MLL-r ALL patients which demonstrated that a subgroup of these patients has no detectable *HOXA* gene expression, while *MEIS1* expression was universally observed [7, 8]. Those patients with reduced *HOXA* gene transcription instead displayed overexpression of the *IRX1* gene which in a cell-based overexpression system was shown to not only decrease the expression level of *HOXA* genes but also that of *MEIS1* [7, 31]. These findings suggest that *MEIS1* expression might vary in MLL-r leukemia patients, which we confirmed in a large pediatric MLL-r ALL patient dataset.

A role for MEIS1 in the regulation of metabolic phenotype and oxidative phosphorylation in hematopoietic stem cells and MLL-AF9 transfected cells has recently been reported [27, 28, 32]. Loss of *MEIS1* in high-MEIS1-expressing leukemia stem cells was associated with increased cellular ROS and heightened oxidative phosphorylation, and knockdown of *MEIS1* in MLL-AF9-driven in vitro and in vivo leukemia models increased oxidative phosphorylation [27, 28, 32]. HIF1α, a well-established transcriptional regulator of hypoxic responses that stimulates cells to switch to anaerobic glycolysis, was thereby identified as the downstream mediator of MEIS1 in regulating metabolic phenotype [27, 33, 34]. It is now postulated that one of the leukemia-driving roles of MEIS1 entails regulating metabolic phenotype through regulating the transcription of HIF1α. Therefore, it is conceivable that MLL-r leukemia cells with lower expression of the MEIS1/HIF1α pathway are less metabolically flexible in their response to specific mitochondrial stress induced by CCI-006, and thus sensitive to the inhibition of mitochondrial respiration induced by the compound. This variable metabolic flexibility to CCI-006-induced respiration inhibition is not due to differences in baseline mitochondrial content and mitochondrial activity and does not correlate with general susceptibility to mitochondrial stressors. Sensitive cells were however characterized by decreased levels of Complex V ATP synthase indicating a differential mitochondrial respiratory chain constitution. As Complex V ATP synthase has been shown to play a role in maintaining membrane potential in conditions of mitochondrial respiration chain dysfunction through an oxidative phosphorylation-independent mechanism, it is possible that cells with higher levels of this complex (such as the unresponsive cells vs. the sensitive cells) may be better equipped to maintain mitochondrial membrane potential when mitochondrial respiration is acutely inhibited, such as upon CCI-006 addition [35].

Based on increasing evidence supporting a role for oxidative respiration in sustaining cancer cell survival and proliferation, the therapeutic targeting of mitochondrial metabolism in cancer, including hematological malignancies such as acute leukemias, has gained renewed interest (reviewed in ref. [36–42]). Several recent studies support the existence of leukemic cell subsets in AML and ALL that are more dependent on mitochondrial respiration than glycolysis [43–47]. This has provided impetus for investigations into the therapeutic potential of targeting mitochondrial function by inhibiting respiratory complexes (such as by rotenone), mitochondrial translation (tigecycline), or the mitochondrial protease ClpP [43–47]. We report here for the first time on a novel compound-mediated inhibition of mitochondrial respiration that selectively induces cell death in a highly aggressive subset of acute MLL-r leukemias.

The identification of the actual target of CCI-006 will help us understand how the compound exactly inhibits respiration. As CCI-006 contains a sulfonamide group and sulfonamides have been described as potent inhibitors of carbonic anhydrases [48], enzymes that perform numerous functions in the cell including roles in mitochondrial respiration [48–50], we investigated the potential of CCI-006 as an inhibitor of these enzymes. We found that CCI-006 was a very potent inhibitor of several carbonic anhydrases in vitro (data not shown). However, commercially available carbonic anhydrase inhibitors were not able to mimic the MLL-r leukemia cell-killing induced by CCI-006, indicating that carbonic anhydrase inhibition is likely not the main or sole mechanism of action of CCI-006. In addition, based on the low stability of the compound in liver microsomal stability assays, CCI-006 is predicted to have insufficient stability to allow in vivo testing (Supplementary Figure 8). Future studies therefore entail focused library screening of CCI-006 analogues to identify a more stable and potent, drug-like molecule derived from CCI-006 that is amenable to target-identification approaches and in vivo efficacy studies.

In conclusion, we have identified a compound that as a single agent selectively kills a subgroup of MLL-r and CALM-AF10 leukemia cells characterized by relatively low-expression levels of HIF1α and MEIS1. Our findings support the presence of metabolic variability within MLL-r leukemia that might partly underlie the postulated heterogeneity of the disease, providing a rationale for the development of therapeutic approaches individually tailored toward specific disease subtypes, in order to achieve significant overall improvement of patient survival rates.

## Materials and methods

### Cells, cell-based screening, cellular assays, generation of CCI-006 resistant cell lines, and siRNA transfection

The panel of cell lines used for experiments is described in Supplementary Table 1. Peripheral blood mononuclear cells from healthy donors were purchased from Australian Red Cross. The culturing of cells, the phenotypic screen, the assays to measure viability, apoptosis and mitochondrial membrane potential, and the generation of resistant cell lines were performed as previously described [10]. In synergy assays, cells were exposed to a dilution series of compounds as single agent and in combination in resazurin reduction assays and the occurrence of synergy was determined with the Bliss Independence model [14, 15]. For EPZ-5676, cells were pre-treated for 7 (PER-485) or 10 (U937) days prior to addition of CCI-006 for 3 days as previously described for synergy studies with EPZ-5676 [51]. Cells were transfected with HIF1A siRNA (100 nM, FlexiTube siRNA, 1:1 of SI02664431 and SI02664053 duplexes, Qiagen, Chadstone, Victoria, Australia) and control siRNA (ON-TARGETplus Non-Targeting Pool, Dharmacon, Lafayette, CO, USA) complexed to star-POEGMA nanoparticles (4:1 (w/w) ratio with siRNA) as previously described [23]. After 18 h, cells were harvested for immunoblotting and treatment with 12.5 µM CCI-006 or vehicle for 6 h. Cell viability was subsequently assessed through live cell counting by Trypan blue exclusion assay.

### Protein and RNA analysis

Assays were performed as previously described [10]. Primers, probes and antibodies are described in Supplementary Tables 3 and 4, respectively. For polysome profiling, 10–20 million cells were treated with 5 μM CCI-006 or vehicle for 3 h, followed by a treatment with 50 µg/ml cycloheximide for 15 min prior to harvesting, washing and lysing in hypotonic wash and lysis buffer (Supplementary Table 5), respectively. Lysates equivalent to equal cell number were separated on 10–40% sucrose gradients (ICSO Model 160 Gradient Former, Teledyne, Thousand Oaks, CA, USA), then fractionated using an ISCO UA-6 UV/VIS detector (Teledyne).

### Citrate synthase activity measurement

Citrate synthase activity was determined in cell pellets as previously described [52, 53].

### Respiration measurements

Liver mitochondria from 129S1/SvImJ mice (*n* = 5) were isolated as described [54]. Respiration measurements in isolated liver mitochondria and cell lines were conducted in a Clark-type oxygen electrode (Rank Brothers, Cambridge, UK). Media used are described in Supplementary Table 5. Following attainment of a steady basal rate of oxygen consumption, 1 μM CCI-006 was added. For ADP-stimulated mitochondria, 0.2 mM ADP was added to the chamber and 5 µM CCI-006 was added after achieving State III respiration. Mitochondrial respiration data are presented as nanomoles of oxygen consumed per minute per mg protein. Cellular data are presented as percentage oxygen consumption rate compared to baseline readings prior to compound administration. Cells were measured in duplicate over two independent experimental days. Rates were the average of two to four individual rate measurements per condition per replicate.

### Metabolite labeling assays

Cells were labeled for 24 h in MEM/NEAA medium as previously described [55], using 50% U-^13^C6-glucose as the labeling substrate. Cells were extracted, and analyzed by gas chromatography–mass spectrometry as previously described [55].

### Mouse liver microsomal stability assays

Reaction mixtures containing potassium phosphate buffer (pH 7.4) and NADPH Regenerating System Solution A and B (BD Biosciences, North Ryde, New South Wales, Australia) were prepared according to supplier specifications and pre-incubated at 37 °C. Test compounds dissolved in DMSO (1 μM) were added to liver homogenates (0.5 mg/m) and incubated at 37 °C. At each time point (0, 5, 10, 15, 30, 60, and 90 min), 70 μL of assay mix was extracted and added to 70 μL acetonitrile (on ice). Supernatant from centrifuged (10,000*g*, 3 min) samples was analyzed for presence of CCI-006 by mass spectrometry at the Bioanalytical Mass Spectrometry Facility, UNSW, Sydney, Australia. Compound half-life was estimated by nonlinear regression.

### Microarray and gene-expression analysis

Microarray-based gene-expression experiments were performed as previously described [10] and microarray data were deposited (Accession code GSE115833; https://www.ncbi.nlm.nih.gov/geo/query/acc.cgi?acc=GSE115833).

### Statistical analysis

Statistical analyses were performed with GraphPad Prism7 Software. Statistical significance of mean differences between groups was determined by Student’s *t* test, ANOVA followed by Tukey’s multiple comparison test or one sample *t* test. The significance of associations between variables was determined by Pearson’s correlation coefficient. A two-sided *P* value < 0.05 was considered statistically significant.

## Supplementary information

Supplemental Information
