# Supplementary material for: A novel small molecule that kills a subset of MLL-rearranged leukemia cells by inducing mitochondrial dysfunction
Source: Oncogene. 2019 Jan 22;38(20):3824–42. doi: 10.1038/s41388-018-0666-5 (PMC6756102; doi:10.1038/s41388-018-0666-5)
Supplement: Supplementary file 1 — Supplemental Information [file 41388_2018_666_MOESM1_ESM.docx]

**SUPPLEMENTARY INFORMATION**

**SUPPLEMENTARY FIGURES**

**Supplementary Figure 1: CCI-006 induces a pro-apoptotic UPR in CALM-AF10 leukemia cells**


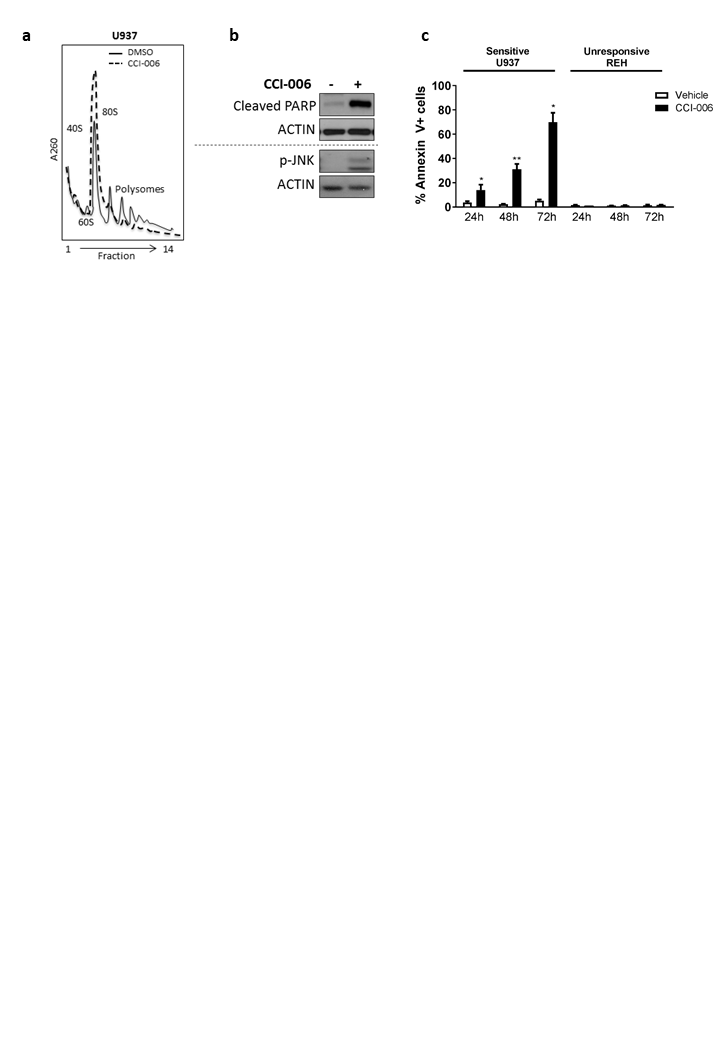


**(a)** Representative result of polysome profiling experiments performed on CALM-AF10 U937 cells (n=2 independent experiments) treated with 5 μM CCI-006 and vehicle for 3h. **(b)** Western blotting for cleaved PARP and phosphorylated JNK on lysates from U937 cells treated with 5 μM CCI-006 or vehicle for 3h (p-JNK) and 24h (cleaved PARP). Blot is representative of 2 independent experiments. **(c)** CCI-006 induces significant increases in the percentage of Annexin V-positive cells in U937 cells treated with 10 μM CCI-006 for 24h (n=3 independent experiments), 48h (n=3) and 72h (n=2) while such an effect was absent in unresponsive REH cells (n=2). Bar graph shows mean ± SEM of the percentage of Annexin V-positive apoptotic cells as analysed by flow cytometry. Mean percentages of Annexin V-positive cells in CCI-006-treated and vehicle-treated cells were compared by t-tests. *, P<0.05; **, P<0.01.

**Supplementary figure 2: Characteristics of induced and acquired resistance to CCI-006**

**
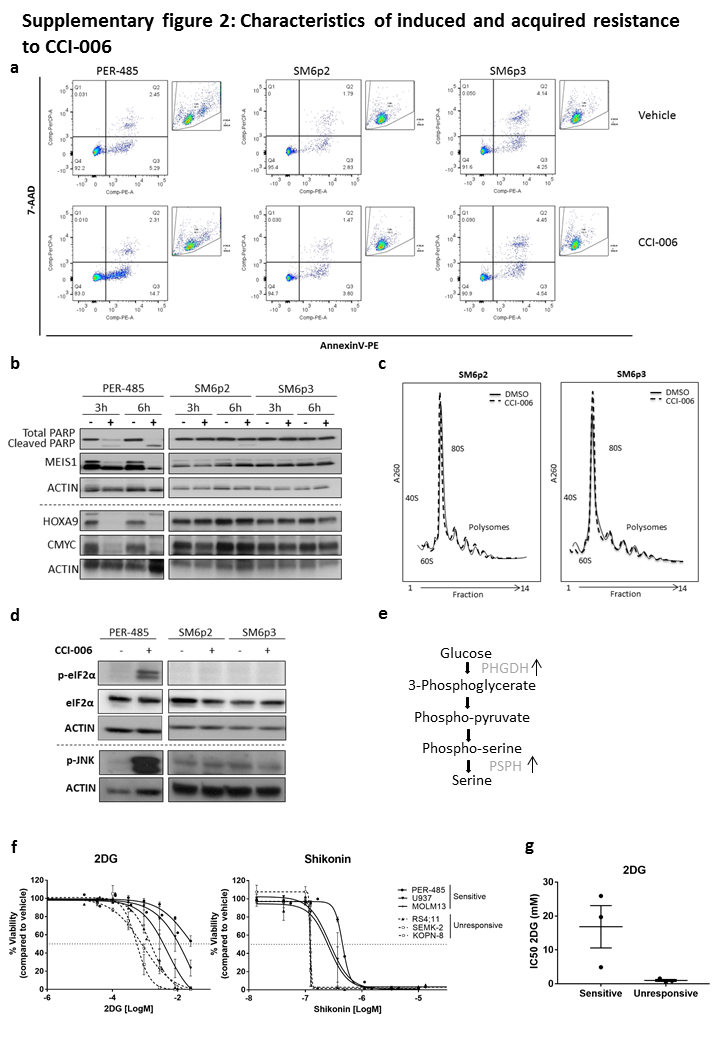
**

**(a)** Representative AnnexinV-PE and 7-AAD FACS staining of PER-485, SM6p2 and SM6p3 cells treated with 5 μM CCI-006 or vehicle for 3h (n=2 independent experiments). **(b)** Representative Western blot of PER-485, SM6p2 and SM6p3 cells treated with 5 μM CCI-006 or vehicle for 3h and 6h (n=2 independent experiments). **(c)** Representative result of polysome profiling experiments performed on SM6p2 and SM6p3 cells (n=2 independent experiments) treated with 5 μM CCI-006 ~~(+)~~ and vehicle ~~(-)~~ for 3h. **(d)** Western blot of PER-485, SM6p2 and SM6p3 cells treated with 5 μM CCI-006 (+) or vehicle (-) for 3h. **(e)** Schematic representation of serine biosynthesis pathway with indication of enzymes for which differential gene expression was detected in SM6p3/SM6p2 versus PER-485 cells. PSPH, phosphoserine phosphatase; PHGDH, phosphoglycerate dehydrogenase. **(f)** Viability of CCI-006-sensitive (PER-485, MOLM13, U937) and unresponsive (RS4;11, KOPN-8, SEMK-2) leukemia cells upon incubation with a dose-range of 2DG or shikonin for 72h as evaluated with a resazurin-based assay. Each data point represents mean % viability (relative to vehicle-treated cells) ± SEM of at least 2 independent experiments. **(g)** Corresponding mean IC50 values ± SEM determined based on 72h viability assays in (f). Mean IC50s per group were compared by t-test but were not significantly different.

**Supplementary Figure 3: GSEA identified enriched pathways in SM6p3 versus PER-485 cells**

**
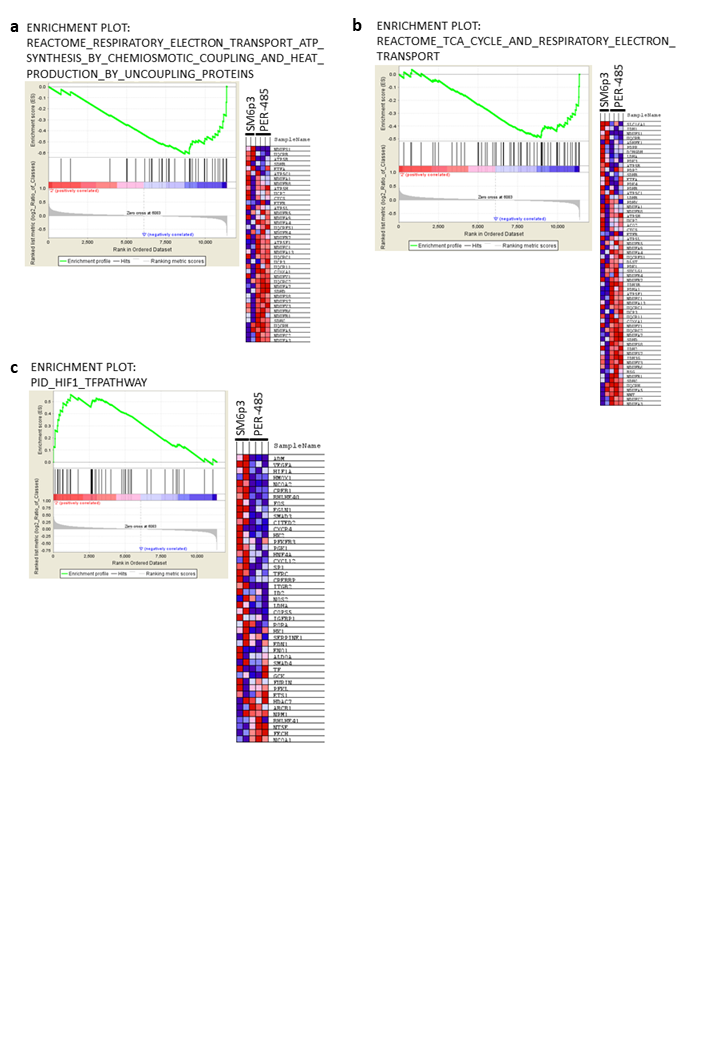
**

**(a)** Enriched in PER-485 cells versus SM6p3 cells.: NES: -1.668, FDR: 0.1185. **(b)** Enriched in PER-485 cells versus SM6p3 cells: NES: -1.446, FDR: 0.2331. **(c)** Enriched in SM6p3 cells versus PER-485 cells: NES: 1.682, FDR: 0.0996.

**Supplementary Figure 4: CCI-006 affects mitochondrial functioning and mitochondrial membrane potential**

**
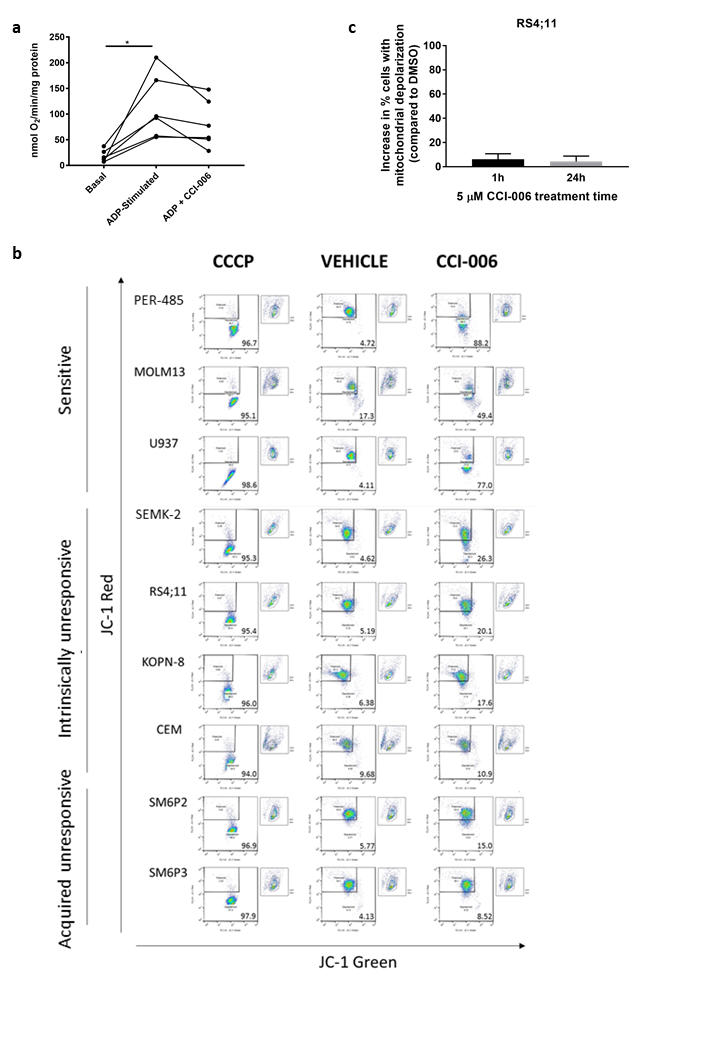
**

**(a)** Effect of 5 μM CCI-006 addition on ADP-stimulated oxygen consumption by isolated mouse liver mitochondria (n=5). Mean oxygen consumptions between unstimulated versus ADP-stimulated mitochondria and ADP-stimulated untreated versus ADP-stimulated CCI-006-treated mitochondria were compared by paired t-test. **(b)** Representative JC-1 staining of a panel of sensitive and unresponsive cells treated for 1h with 5 μM CCI-006 or vehicle, as measured by flow cytometry. An increase in the percentage of cells with depolarized mitochondria is indicated by a shift in JC-1 red to green signal. Dot blots show representative data for 3 independent experiments. Treatment with uncoupler carbonyl cyanide m-chlorophenyl hydrazone (CCCP) was included as a positive control for induction of mitochondrial membrane depolarization. **(c)** Mean increase in percentage of RS4;11 cells with depolarized mitochondria after 1h and 24h treatment with 5 μM CCI-006 compared to vehicle-treated cells (n=2). *, P<0.05.

**Supplementary Figure 5: Comparison of CCI-006 sensitive and unresponsive MLL-r and CALM-AF10 leukemia cells**

**
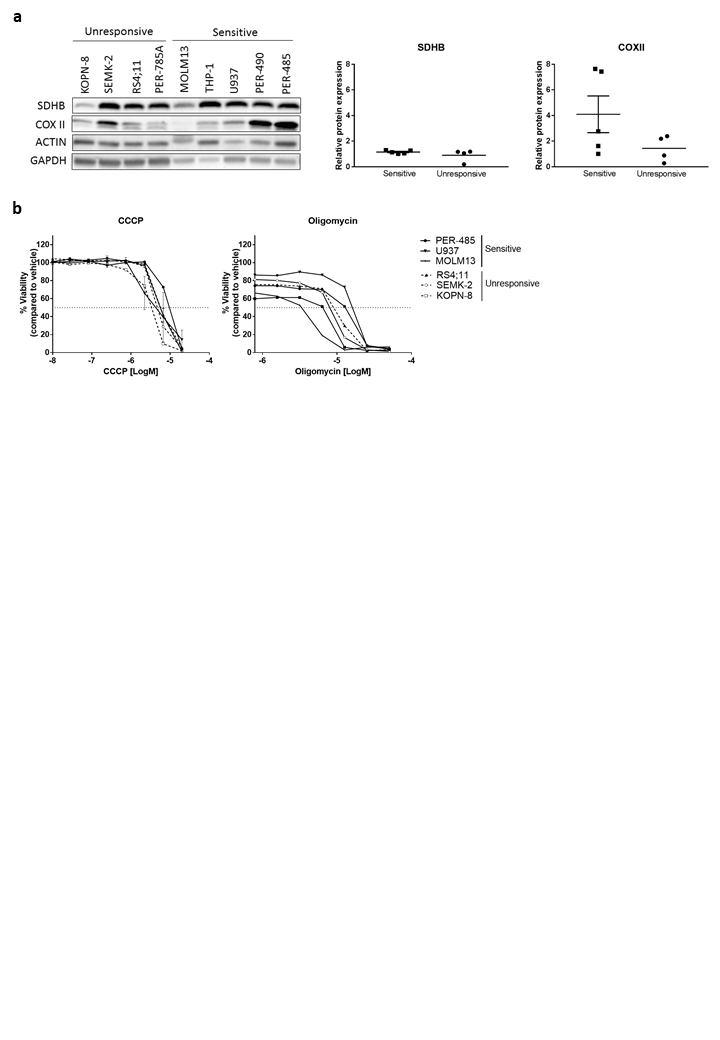
**

**(a)** Representative blot for Mitochondrial Complex II SDHB and Mitochondrial Complex IV COXII in a panel of unresponsive and sensitive MLL-r and CALM-AF10 leukemia cells. Densitometry was performed on blots performed on 3 (SDHB, GAPDH) or 2 (COXII) independent cell harvests and protein expressions were normalized to GAPDH, followed by normalization across cell lines to PER-485 cells. Dot points represent the mean relative protein expression of a cell line. The mean relative protein expressions between sensitive and unresponsive cells were compared by t-test. **(b)** Resazurin reduction-based viability assays of CCI-006-sensitive (PER-485, MOLM13, U937) and unresponsive (RS4;11, KOPN-8, SEMK2) leukemia cells incubated with mitochondrial stressors CCCP and oligomycin. Each data point represents mean % viability (relative to vehicle-treated cells) ± SEM of at least 2 independent experiments.

**Supplementary Figure 6: Messenger RNA expression levels of *MEIS1* and *ATP5I* in the Murphy database**

**
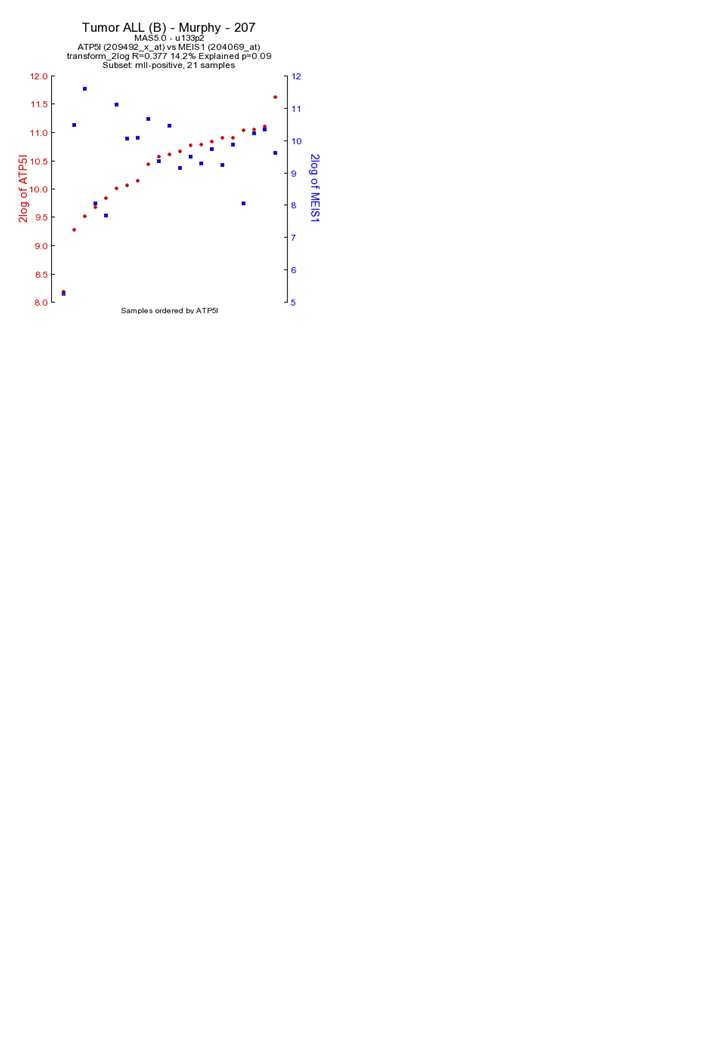
**

Messenger RNA expression levels of *MEIS1* and *ATP5I* in the Murphy database (n=21 MLL-r leukemia patients) as analysed through R2.

**Supplementary figure 7: CCI-006 and CCI-007 decrease the survival of MLL-r leukemia cells in different ways**


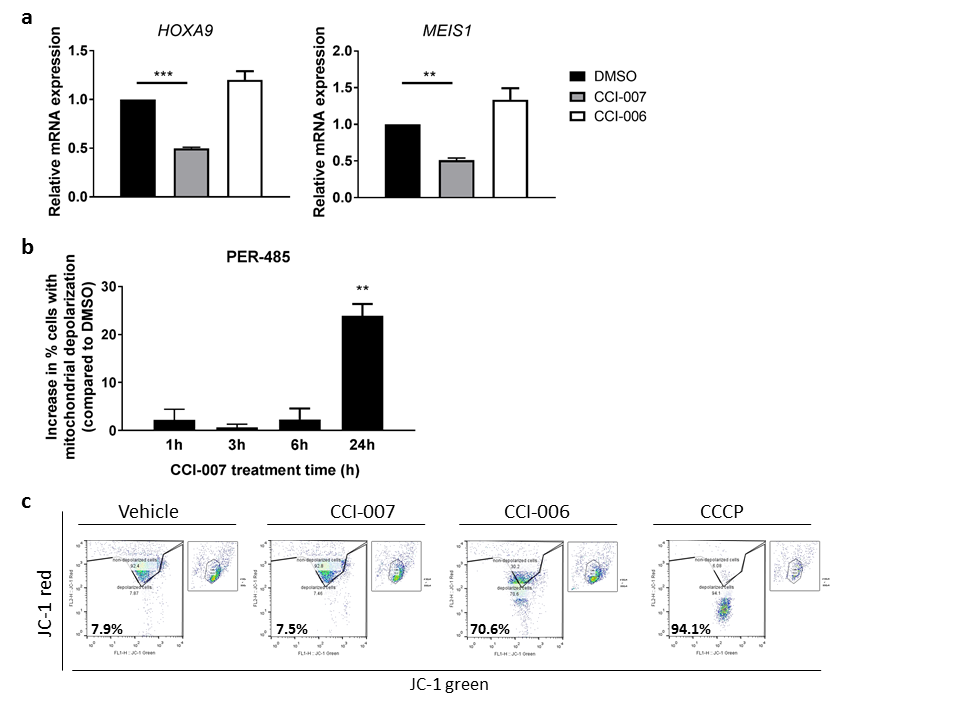


**(a)** *HOXA9* and *MEIS1* mRNA levels were assayed in PER-485 cells treated with 5 µM CCI-006, CCI-007 or vehicle (DMSO) for 3h by quantitative RT-PCR and relative expressions were calculated using the ΔΔCt method. Gene expressions were normalized against housekeeping genes and expressed relative to vehicle-treated cells. Each data point represents the mean + SEM of 3 independent experiments. Mean relative expressions were compared between treated and vehicle-treated cells by one-sample t-tests. **(b)** Increase in the percentage of PER-485 cells with depolarized mitochondria after incubation with 5 µM CCI-007 up to 24h compared to vehicle-treated cells. Bar graphs represent the mean of at least 2 independent experiments + SEM. T-tests were used to compare the mean percentage of cells with depolarized mitochondria in CCI-007 versus vehicle-treated cells. **(c)** JC-1 staining of PER-485 cells treated with 5 µM CCI-006, CCI-007 or vehicle (DMSO) in the same experiment. An increase in the number of cells with mitochondrial membrane depolarization is evidenced by a shift in JC-1 red to green signal for the cell population. Treatment with uncoupler carbonyl cyanide m-chlorophenyl hydrazone (CCCP) was included as a positive control for induction of mitochondrial membrane depolarization. Displayed percentage (%) in dot plots represents the percentage of cells with depolarized mitochondria. **, P<0.01; ***, P<0.001.

**Supplementary Figure 8: CCI-006 has limited stability in mouse liver microsomal stability assays**

**
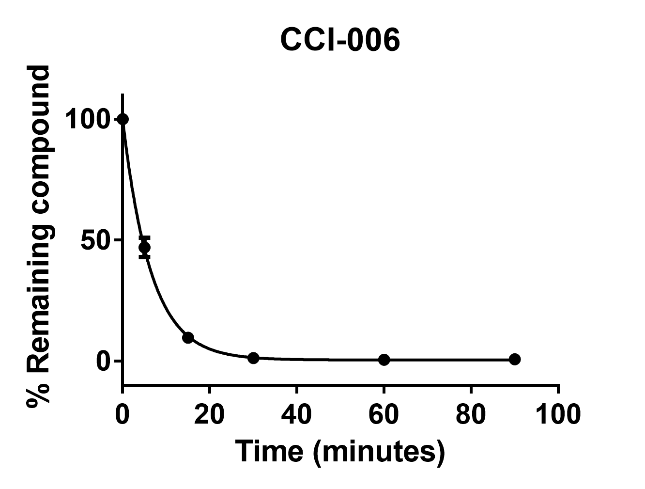
**

Non-linear regression fit of microsomal stability data of CCI-006. Each point represents the mean of 4 independent measurements at a certain time point. CCI-006 has an estimated half-life of 4.5 minutes in mouse liver microsomal stability assays.

**SUPPLEMENTARY TABLES**

**Supplementary Table 1: Cell line characteristics**

| **Cell Line** | **MLL status** | **Disease** | | **Supplier** | **Authentication^1,2^** |
| --- | --- | --- | --- | --- | --- |
|  |  |  | |  |  |
| PER-485 | t(4;11) | Infant ALL | | Kees UR et al. Mol Cancer Ther 2003 | STR profiling 22/03/2016 |
| PER-490 | t(4;11) | Infant ALL | | Kees UR et al. Mol Cancer Ther 2003 | STR profiling 22/03/2016 |
| PER-703A | t(1;11) | Infant ALL | | Kees UR et al. Mol Cancer Ther 2003 | STR profiling 22/03/2016 |
| PER-785A | t(4;11) | Infant ALL | | Kees UR et al. Mol Cancer Ther 2003 | STR profiling 22/03/2016 |
| PER-826A | complex t(11;19) | Infant ALL | | Kees UR et al. Mol Cancer Ther 2003 | STR profiling 22/03/2016 |
| MV4;11 | t(4;11) | Childhood AML | | ATCC | STR profiling 05/07/2013 |
| RS4;11 | t(4;11) | Pre-B cell ALL | | ATCC | STR profiling 22/03/2016 |
| SEMK-2 | t(4;11) | Pre-B cell childhood ALL | | ATCC | STR profiling 05/07/2013 |
| MOLM13 | t(9;11) | AML | | R D’Andrea, IMVS, South Australia, Australia | STR profiling 15/08/2015 |
| THP-1 | t(9;11) | Infant AML | | W Jessup, Centre for Vascular Research, NSW, Australia | STR profiling 22/03/2016 |
| KOPN-8 | t(11;19) | Infant pre-B ALL | | DSMZ | STR profiling 22/03/2016 |
| U937 | MLL-wt CALM-AF10 | AML derived from histiocytic lymphoma | | ATCC | STR profiling 17/06/2014 |
| KP-MO-TS | MLL-wt CALM-AF10 | AML | | Imamura T et al. Leukemia 2002 | NA^3^ |
| Loucy | MLL-wt SET-NUP214 | T-cell ALL | | ATCC | STR profiling 22/03/2016 |
| KG-1 | MLL-wt | AML | | ATCC | STR profiling 22/03/2016 |
| Kasumi-1 | MLL-wt | AML | | ATCC | STR profiling 22/03/2016 |
| REH | MLL-wt | Pre-B cell ALL | | ATCC | STR profiling 22/03/2016 |
| CEM | MLL-wt | T-cell ALL | | ATCC | STR profiling 09/05/2013 |
| K562 | MLL-wt | CML | | ATCC | STR profiling 22/03/2016 |
| Hal-01 | MLL-wt | ALL | | A Thomas, Dana-Farber Cancer Institute, Massachusetts, USA | STR profiling 22/03/2016 |
| UOC-B1 | MLL-wt | Promyelocytic leukemia | | A Thomas, Dana-Farber Cancer Institute, Massachusetts, USA | STR profiling 22/03/2016 |
| NB4 | MLL-wt | Acute promyelocytic leukemia | | ATCC | STR profiling 22/03/2016 |
| Calu-6 | MLL-wt | Lung carcinoma | | ATCC | STR profiling 22/03/2016 |
| MCF-7 | MLL-wt | Breast adenocarcinoma | | ATCC | STR profiling 22/03/2016 |
| HeLa | MLL-wt | Adenocarcinoma | | ATCC | STR profiling 31/07/2014 |
| BE(2)-C | MLL-wt | Neuroblastoma | | J Biedler, Memorial Sloan-Kettering Cancer Centre, NY, USA | STR profiling 04/12/2012 |
| HSF | MLL-wt | Primary skin fibroblast cells | | ATCC | STR profiling 22/03/2016 |
| MRC5 | MLL-wt | Lung fibroblast | | ATCC | STR profiling 22/03/2016 |
| MCF10a | MLL-wt | Epithelial cell | | ATCC | STR profiling 22/03/2016 |
| ^1^ Method and date of authentication | | |  |  |  |
| ^2^ All cell stocks are tested for mycoplasma contamination and cells in culture are tested every 6 months  ^3^ Cells up to ten passages from the original stock were used for experiments | | | | | |

**Supplementary Table 2: Gene Annotation Enrichment Analysis: differentially expressed genes between PER-485 and SM6p3 in KEGG pathways associated with cellular metabolism**

| **KEGG pathway** | **Genes** |
| --- | --- |
| Biosynthesis of antibiotics | NME1-NME2 readthrough(NME1-NME2) |
|  | UDP-glucose pyrophosphorylase 2(UGP2) |
|  | adenylate kinase 1(AK1) |
|  | adenylate kinase 4(AK4) |
|  | alcohol dehydrogenase 5 (class III), chi polypeptide(ADH5) |
|  | aldehyde dehydrogenase 3 family member A2(ALDH3A2) |
|  | aldolase, fructose-bisphosphate C(ALDOC) |
|  | farnesyltransferase, CAAX box, alpha(FNTA) |
|  | fructose-bisphosphatase 1(FBP1) |
|  | galactose mutarotase(GALM) |
|  | lactate dehydrogenase A like 6A(LDHAL6A) |
|  | methylsterol monooxygenase 1(MSMO1) |
|  | phosphoribosyl pyrophosphate amidotransferase(PPAT) |
|  | phosphoribosyl pyrophosphate synthetase 1-like 1(PRPS1L1) |
|  | aminomethyltransferase(AMT) |
|  | argininosuccinate synthase 1(ASS1) |
|  | farnesyl diphosphate synthase(FDPS) |
|  | phosphogluconate dehydrogenase(PGD) |
|  | phosphoserine phosphatase(PSPH) |
|  | serine dehydratase like(SDSL) |
|  | succinate dehydrogenase complex subunit C(SDHC) |
|  | phosphoglycerate dehydrogenase(PHGDH) |
| Carbon metabolism | acyl-CoA dehydrogenase, C-2 to C-3 short chain(ACADS) |
|  | alcohol dehydrogenase 5 (class III), chi polypeptide(ADH5) |
|  | aldolase, fructose-bisphosphate C(ALDOC) |
|  | aminomethyltransferase(AMT) |
|  | carbamoyl-phosphate synthase 1(CPS1) |
|  | fructose-bisphosphatase 1(FBP1) |
|  | phosphogluconate dehydrogenase(PGD) |
|  | phosphoglycerate dehydrogenase(PHGDH) |
|  | phosphoribosyl pyrophosphate synthetase 1-like 1(PRPS1L1) |
|  | phosphoserine phosphatase(PSPH) |
|  | serine dehydratase like(SDSL) |
|  | succinate dehydrogenase complex subunit C(SDHC) |
| Metabolic pathways | N-acetyltransferas+RC:R[39]Ce 1(NAT1) |
|  | NADH:ubiquinone oxidoreductase subunit A3(NDUFA3) |
|  | NADH:ubiquinone oxidoreductase subunit C2(NDUFC2) |
|  | NME1-NME2 readthrough(NME1-NME2) |
|  | UDP-GlcNAc:betaGal beta-1,3-N-acetylglucosaminyltransferase 2(B3GNT2) |
|  | UDP-glucose pyrophosphorylase 2(UGP2) |
|  | acyl-CoA dehydrogenase family member 8(ACAD8) |
|  | acyl-CoA dehydrogenase, C-2 to C-3 short chain(ACADS) |
|  | acyl-CoA synthetase long-chain family member 3(ACSL3) |
|  | acyl-CoA thioesterase 4(ACOT4) |
|  | adenosine kinase(ADK) |
|  | adenylate kinase 1(AK1) |
|  | adenylate kinase 4(AK4) |
|  | alcohol dehydrogenase 5 (class III), chi polypeptide(ADH5) |
|  | aldehyde dehydrogenase 3 family member A2(ALDH3A2) |
|  | aldolase, fructose-bisphosphate C(ALDOC) |
|  | aminomethyltransferase(AMT) |
|  | arachidonate 5-lipoxygenase(ALOX5) |
|  | argininosuccinate synthase 1(ASS1) |
|  | arylsulfatase B(ARSB) |
|  | asparagine synthetase (glutamine-hydrolyzing)(ASNS) |
|  | carbamoyl-phosphate synthase 1(CPS1) |
|  | ubiquinol-cytochrome c reductase hinge protein(UQCRH) |
|  | ubiquinol-cytochrome c reductase, complex III subunit X(UQCR10) |
|  | xylosyltransferase 1(XYLT1) |
|  | cat eye syndrome chromosome region, candidate 1(CECR1) |
|  | catechol-O-methyltransferase(COMT) |
|  | creatine kinase B(CKB) |
|  | creatine kinase, M-type(CKM) |
|  | dehydrogenase/reductase 9(DHRS9) |
|  | farnesyl diphosphate synthase(FDPS) |
|  | fructose-bisphosphatase 1(FBP1) |
|  | galactose mutarotase(GALM) |
|  | glycerol-3-phosphate acyltransferase 3(GPAT3) |
|  | heparan-alpha-glucosaminide N-acetyltransferase(HGSNAT) |
|  | lactate dehydrogenase A like 6A(LDHAL6A) |
|  | methylsterol monooxygenase 1(MSMO1) |
|  | nicotinamide nucleotide transhydrogenase(NNT) |
|  | phosphogluconate dehydrogenase(PGD) |
|  | phosphoglycerate dehydrogenase(PHGDH) |
|  | phospholipase A2 group IIC(PLA2G2C) |
|  | phospholipase D family member 3(PLD3) |
|  | phosphoribosyl pyrophosphate amidotransferase(PPAT) |
|  | phosphoribosyl pyrophosphate synthetase 1-like 1(PRPS1L1) |
|  | phosphoserine phosphatase(PSPH) |
|  | primase (DNA) subunit 2(PRIM2) |
|  | ribonucleotide reductase regulatory subunit M2(RRM2) |
|  | serine dehydratase like(SDSL) |
|  | succinate dehydrogenase complex subunit C(SDHC) |
|  | thiamin pyrophosphokinase 1(TPK1) |
| Biosynthesis of amino acids | aldolase, fructose-bisphosphate C(ALDOC) |
|  | argininosuccinate synthase 1(ASS1) |
|  | carbamoyl-phosphate synthase 1(CPS1) |
|  | phosphoglycerate dehydrogenase(PHGDH) |
|  | phosphoribosyl pyrophosphate synthetase 1-like 1(PRPS1L1) |
|  | phosphoserine phosphatase(PSPH) |
|  | serine dehydratase like(SDSL) |
| Glycolysis / Gluconeogenesis | alcohol dehydrogenase 5 (class III), chi polypeptide(ADH5) |
|  | aldehyde dehydrogenase 3 family member A2(ALDH3A2) |
|  | aldolase, fructose-bisphosphate C(ALDOC) |
|  | fructose-bisphosphatase 1(FBP1) |
|  | galactose mutarotase(GALM) |
|  | lactate dehydrogenase A like 6A(LDHAL6A) |
| Pentose phosphate pathway | aldolase, fructose-bisphosphate C(ALDOC) |
|  | fructose-bisphosphatase 1(FBP1) |
|  | phosphogluconate dehydrogenase(PGD) |
|  | phosphoribosyl pyrophosphate synthetase 1-like 1(PRPS1L1) |
| Alanine, aspartate and glutamate metabolism | argininosuccinate synthase 1(ASS1) |
|  | asparagine synthetase (glutamine-hydrolyzing)(ASNS) |
|  | phosphoribosyl pyrophosphate amidotransferase(PPAT) |
|  | carbamoyl-phosphate synthase 1(CPS1) |
|  |  |

**Supplementary Table 3: Antibodies**

| **Antibody** | **Catalogue number/Clone** | **Supplier** |
| --- | --- | --- |
|  |  |  |
| rabbit anti-HOXA9 | 07-178 | Merck Millipore, Bayswater, Victoria, Australia |
| rabbit anti-MEIS1 | NBP1-95898/EPR5781 | Novus Biologicals, In Vitro Technologies, Noble Park North, Victoria, Australia |
| rabbit anti-CMYC | 9402 | Cell Signaling Technology, Genesearch, Arundel, Queensland, Australia |
| rabbit anti-cleaved PARP | 5625 | Cell Signaling Technology |
| mouse anti-PARP | sc-53643/C2-10 | Santa Cruz Biotechnology, Dallas, Texas |
| rabbit anti-cleaved CASPASE-3 | 9664 | Cell Signaling Technology |
| anti-CASPASE 3 | 9662 | Cell Signaling Technology |
| rabbit anti-phospho-eIF2α (Ser51) | 9721 | Cell Signaling Technology |
| rabbit anti-eIF2α | 9722 | Cell Signaling Technology |
| mouse anti-phospho-SAPK/JNK | 9255 | Cell Signaling Technology |
| rabbit anti-HIF1α | ab2185 | Abcam, Cambridge, MA, USA |
| Total OXPHOS Human WB Antibody Cocktail | ab110411 | Abcam |
| rabbit anti-VDAC | 4866 | Cell Signaling Technology |
| mouse anti-GAPDH | G8795 | Sigma-Aldrich |
| rabbit anti-ACTIN | A2668 | Sigma-Aldrich |

**Supplementary Table 4: Primers and probes**

| **Primer/Probe** | **Sequence/Catalogue number** | **Supplier** |
| --- | --- | --- |
|  |  |  |
| HOXA9 FORWARD | 5’GAC AAG CCC CCC ATC GAT 3’ | Sigma-Aldrich |
| HOXA9 REVERSE | 5’ GAG TGG AGC GCG CAT GA 3’ | Sigma-Aldrich |
| MEIS1 FORWARD | 5’ TCG CGC AGA AAA ACC TCT ATT 3’ | Sigma-Aldrich |
| MEIS1 REVERSE | 5’ TTG TCA CAT AAT TCG TGT ACC 3’ | Sigma-Aldrich |
| CHOP | Hs00358796_g1 | Life Technologies |
| GUSB | 4326320E | Life Technologies |
| HPRT | 4326321E | Life Technologies |

**Supplementary Table 5: Buffers and media used for polysome profiling and respiration measurements**

| **Buffer/Media** | **Composition** | **Supplier** |
| --- | --- | --- |
|  |  |  |
| Polysome hypotonic wash buffer | 1.5 mM KCl |  |
|  | 2.5 mM MgCl2 |  |
|  | 5 mM Tris pH 7.5 |  |
|  |  |  |
| Polysome hypotonic lysis buffer | 1.5 mM KCl |  |
|  | 2.5 mM MgCl2 |  |
|  | 0.5% (v/v) Triton X-100 |  |
|  | 0.5% (w/v) sodium deoxycholate | Sigma-Aldrich, Castle Hill, New South Wales, Australia |
|  | EDTA-free Protease inhibitor | Roche, Dee Why, New South Wales, Australia |
|  | 50 μg/ml cycloheximide | Sigma-Aldrich |
|  | 3 mM DTT | Sigma-Aldrich |
|  | 120 U/ml RNasin | Promega, Sydney, New South Wales, Australia |
|  | 5 mM Tris pH 7.5 |  |
|  |  |  |
| Mitochondrial respiration medium | 225 mM mannitol |  |
|  | 75 mM sucrose |  |
|  | 10 mM Tris-HCl |  |
|  | 10 mM KH2PO4 |  |
|  | 10 mM KCl |  |
|  | 0.8 mM MgCl2 |  |
|  | 0.1 mM EDTA |  |
|  | 0.3% fatty acid free BSA | Sigma-Aldrich |
|  | 10mM succinate |  |
|  | pH 7.0 |  |
|  |  |  |
| Cellular respiration medium | XF Basal Medium | Seahorse Bioscience, Agilent Technologies, Mulgrave, Victoria, Australia |
|  | 5.5 mM glucose |  |
|  | 10 mM sodium pyruvate |  |
|  | 2mM glutamine |  |
|  | pH 7.4 |  |
